# Novel Variations in Native Ethiopian Goat breeds PRNP Gene and Their Potential Effect on Prion Protein Stability

**DOI:** 10.1038/s41598-020-63874-z

**Published:** 2020-04-24

**Authors:** Eden Yitna Teferedegn, Yalçın Yaman, Cemal Ün

**Affiliations:** 1 Ege University, Department of Biology, Molecular Biology Division, Izmir, Turkey; 2 Department of Biometry and Genetics, Bandırma Sheep Research Institute, Bandırma Balıkesir, Turkey

**Keywords:** Cell biology, Computational biology and bioinformatics, Genetics, Neuroscience, Structural biology

## Abstract

Scrapie is a lethal neurodegenerative disease of sheep and goats caused by the misfolding of the prion protein. Variants such as M142, D145, S146, H154, Q211, and K222 were experimentally found to increase resistance or extend scrapie incubation period in goats. We aimed to identify polymorphisms in the Afar and Arsi-Bale goat breeds of Ethiopia and computationally assess the effect of variants on prion protein stability. In the present study, four non-synonymous novel polymorphisms G67S, W68R, G69D, and R159H in the first octapeptide repeat and the highly conserved C-terminus globular domain of goat PrP were detected. The resistant genotype, S146, was detected in >50% of the present population. The current study population showed a genetic diversity in Ethiopian goat breeds. In the *insilico* analysis, the R68 variant was predicted to increase stability while S67, D69, and H159 decrease the stability of prion protein. The new variants in the octapeptide repeat motif were predicted to decrease amyloidogenicity but H159 increased the hotspot sequence amyloidogenic propensity. These novel variants could be the source of conformational flexibility that may trigger the gain or loss of function by prion protein. Further experimental study is required to depict the actual effects of variants on prion protein stability.

## Introduction

Prion diseases are lethal neurodegenerative disease of humans and animals caused by the misfolding of prion protein^1^. Despite being rare, the diseases are lethal and have resulted in huge animal loss over a course of time in several countries^2^. The oldest prion disease is scrapie that affects small ruminants^3,4^. Though the exact pathomechanism is not yet known, predisposing genetic variants have been documented in different species^5^. A wide range of polymorphisms have been identified in goats either as protective or susceptible alleles. A recent review reported the presence of more than 50 polymorphisms in goat prion protein-coding gene (PRNP)^6^. Among the variants, G127S, M142I, H143R, N146S, R154H, Q222K, and S240P are the most common substitutions worldwide^7,8^. Similarly, R139S in Algeria^9^, A116V in Tanzania^10^, G134E and Q163P in Turkey^11^ and G127A and T193I in Ethiopia^12^ were novel (at the time of the report) substitutions discovered in the respective area of study. Alleles such as M142, D145, S146, H154, Q211, and K222 were experimentally found to either extend scrapie incubation period or increase resistance^8,13–16^.

Experimental studies reported the link between sequence variation in the functional domains of prion protein and scrapie disease development^17,18^. Substitutions in the palindrome and glycine-rich motifs were identified to affect amyloid fibril formation^19^. Although the C terminal globular domain is often involved in the PrP^c^ to PrP^Sc^ conversion process, octapeptide repeat regions are also known to influence the structural dynamics of prion protein. Insertion or deletion of octapeptide repeat for example, induce disease phenotypes^20^.

According to the FAOSTAT 2017 report, 30 million goats are kept for production in Ethiopia^21^. A microsatellite marker study reported the presence of 4 families and 8 goat breeds distributed across the country. Abergel, Afar, Arsi-Bale, Eastern and South Eastern, Gumuz, Highlan, Keffa and Woyto-Gjam are the main indigenous breeds of Ethiopia. The breeds are named after the name for the place where they are geographically distributed. They are reared for meat, milk and skin production for domestic use and commercial purposes. Due to the limited cross breeding with exotic animals, Ethiopian goat breeds are assumed to maintain ancestral genetic background^22^.

Unlike many other countries, prion disease surveillances and exclusive prion gene genotyping were never conducted in sub-Saharan countries. So far, only a few studies reported polymorphisms in the PRNP of native East African goat breeds^10,12,23^. In the present study, we considered Ethiopian native goat breeds (Afar and Arsi-Bale) to identify variants and their potential implication in the prion protein dynamics computationally. Data from experimental and computational pipelines (MINNUS, SDM, and MILAMP data servers) was triangulated to increase the power of the tests used.

## Results

### Polymorphisms

**Figure 1:**
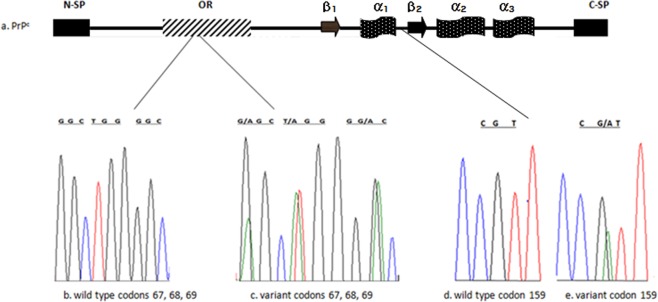
Novel polymorphism in Afar and Arsi-Bale breeds. (**a**) functional domain of prion protein; (**b**) wild type G67, W68 and G69; (**c**) novel variant S67, R68 and D69; (**d**) wild type R159; (**e**) novel variant H159.

In this study, the PRNP coding region of Ethiopian goat breeds from Afar and Arsi-Bale was sequenced and analysed. Four amino acid tandem repeats of P-H-G-G-G-W-G-Q were detected in the population under study. In the first octapeptide repeat, four novel polymorphisms G67G, G67S, W68R, and G69D were detected (Fig. 1). The other novel variant, R159H, was identified in the highly conserved C- terminus globular domain of prion protein (Fig. 1). Along the sequenced region of goat protein-coding gene, 7 additional polymorphic sites were also detected (Table 1). H143R variant was detected at a low frequency in the present study population. The resistant allele N146S was found at a frequency of 0.54 in Arsi-Bale and 0.60 in Afar breeds. The variant R154H was rare in Afar breed. R154H and R159H variants were not detected in Arsi- Bale breed. Some of the previously reported protective variants such as M142, D146 and K222 were not observed. Other known polymorphic codons i.e. I141, G145, R151, P168, R211, and Q222 were detected in the wild type forms in the study population.

**Table 1:** Genotype distribution of variants in native Ethiopian goat breeds.

Amino acid Variants	Codon	Afar N = 35	Arsi-Bale N = 39	P-Value CI = 95%
67 G	GGC	0.51	0.9	0.903
G67G	GGC–>GGT	0.43	0.02	
G67S	GGC–>AGU	0.06	0.08	
68 W	TGG	0.2	0.9	0.019*
W68R	TGG–>AGG	0.8	0.1	
69 G	GGC	0.91	0.92	0.884
G69D	GGC–>GAC	0.09	0.08	
G127	GGC	0.97	0.92	0.837
G127A	GGC–>GCC	0.03	0.08	
S138	AGT	0.71	0.69	0.000*
S138S	AGT–>AGC	0.29	0.31	
H143	CAT	0.97	0.97	0.934
H143R	CAT–>CGT	0.03	0.03	
N146	AAT	0.40	0.46	0.751
N146S	AAT–>AGT	0.60	0.54	
R154	CGT	0.86	1	0.789
R154H	CGT–>CAT	0.14	—	
R159	CGT	0.94	1	0.933
R159H	CGT–>CAT	0.06	—	
T193	ACC	0.94	0.87	0.049*
T193I	ACC–>ATC	0.06	0.13	
Q222	CAG	1	1	
P240	CCC	0.97	0.95	
P240S	CCC–>TCC	0.03	0.05	0.251

*Significant at p < 0.05.

The majority of genotype frequencies were in Hardy Weinberg proportion except at codons 68, 138 and 193. Based on the detected novel and additional polymorphisms in the present study, GWGGSHNRRTP (0.32), GWGGSHNRRTS (0.21) and GRGGSHSRRTS (0.16) were the major haplotypes calculated (Table 2, Supplementary S1).

**Table 2:** Major Haplotypes of polymorphisms in *PRNP* coding sequence of Afar and Arsi-Bale goat breeds of Ethiopia.

	c.199 G > A c.201 C > T	c.202 T > A	c.206 G > A	c.380 G > C	c.414 T > C	c.428 A > G	c.437 A > G	c.461 G > A	c.476 G > A	c.578 C > T	c.718 T > C	
Haplotypes	G67S, G67G	W 68R	G69S	G127A	S138S	H143R	N146S	R154H	R159H	T193I	S240P	N = 100
Hap1	G	W	G	G	S	H	N	R	R	T	P	0.32
Hap2	G	W	G	G	S	H	N	R	R	T	S	0.21
Hap3	G	R	G	G	S	H	S	R	R	T	S	0.16
Hap4	G	W	G	G	S	H	N	H	R	T	P	0.09
Hap5	G	R	G	G	S	H	N	R	R	T	P	0.07
Hap6	S	R	D	G	S	H	N	R	R	T	P	0.02
Hap7	G	W	G	G	S	H	N	R	R	I	P	0.02
Hap8	G	R	G	G	S	H	S	R	R	T	P	0.02
Hap9	G	R	G	G	S	H	S	R	H	T	P	0.02
Hap10	G	W	G	G	S	R	N	R	R	T	P	0.02
Hap11	S	R	G	G	S	H	S	R	R	T	P	0.01
Hap12	S	R	G	G	S	H	N	H	R	T	P	0.01
Hap13	S	R	D	A	S	H	N	R	R	T	P	0.01
Hap14	G	R	G	G	S	H	N	R	R	I	P	0.01
Hap15	G	W	G	G	S	H	N	H	R	T	P	0.01

N = number of haplotypes; >0.9 postprobability score.

### In silico goat prion protein structural dynamics

**Figure 2:**
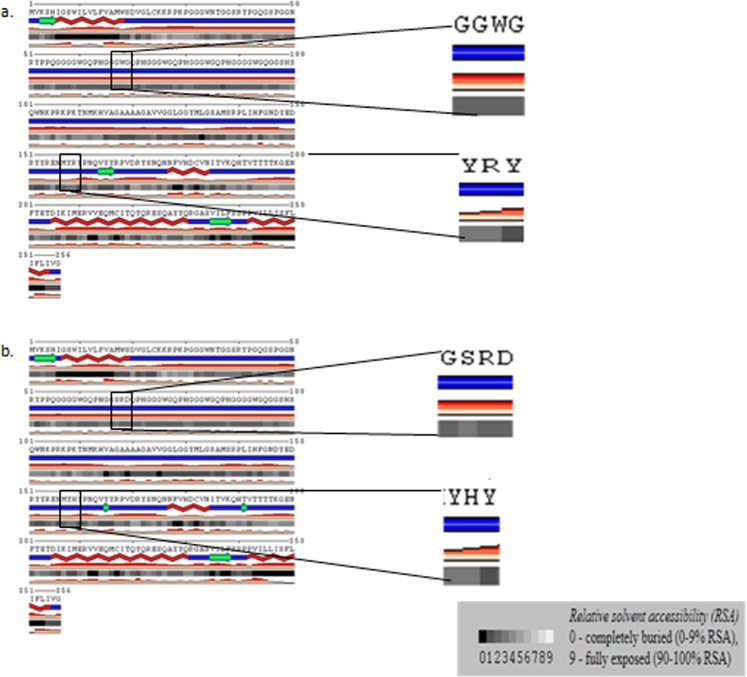
Relative solvent accessibility of Afar and Arsi-Bale goat breeds prion protein with wild type and new varint. Relative solvent accessibility of the:- (**a**) wildtype and (**b**) the new varinat was indicted in a zoomed in pannel. A scale of 0–9 as a measure of surface exposure to solvent at the right lower corner of the pannel. RSA is the net change at the mutated position in the side chain surface accessible area. Lower red blocks indicate confidence level.

**Figure 3:**
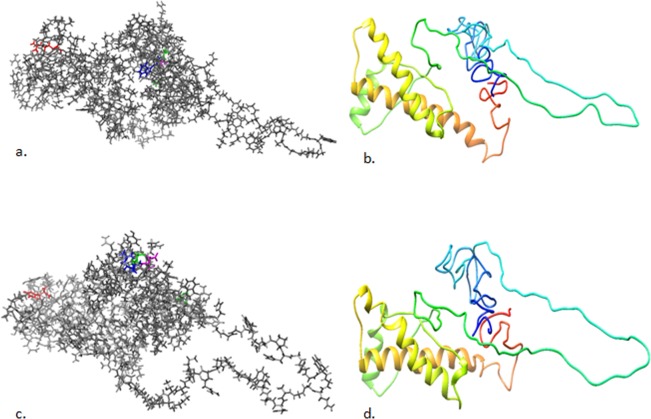
Predicted 3D structure of Afar and Arsi-Bale goat breeds prion protein -UniProt- P52113. (**a**) wild type: G67 green, W68 blue, G69 magenta and R159 red; (**b**) 3DWild type:- C-score = −3.24, Estimated TM-score = 0.35 ± 0.12, Estimated RMSD = 13.6 ± 4.0 Å; (**c**) variant containing structure: S67 green, R68 blue, D69 magenta and HR159 red; (**d**) 3D mutant structure:-C-score = −3.20, Estimated TM-score = 0.36 ± 0.12, Estimated RMSD = 13.5 ± 4.0 Å.

*Insilico* proteins models are effective in identifying mutant substitutions and determining the effect of mutation on prion stability^24^. Using more than one pipelines could increase the power of the tests used in reducing false positives and biases. Here, protein stability in terms of free energy change based on relative solvent accessibility of variants (at the variant position and absolute net change between the wild-type and variant at solvent exposed motif of the protein), and depth (the average distance of all atom depths found in the residue from the nearest surface water molecule) was predicted using computational tools. POLYVIEW-2D prediction platform was used to assess the relative solvent accessibility (RSA) which is based on the degree of surface exposureof the region at the variant position. In the present study, S67 was predicted to increase RSA whereas R68, D69 and H159 rather seem to have neutral effect in terms of solvent accessibility at the mutated position in the side chain surface accessible area (Fig. 2). The effect of variant S67 and R69 on the relative RSA values at solvent exposed regions of the protein was predicted to be neutral (Table 3). The protein structure with substituted variants predicted to be structurally different from the wild type (Fig. 3a,c). In the region between amino acid 155 and 200, the wild type structure consisted of an extended beta sheet (Fig. 3b,d) while the mutant structure comprised of two short beta sheets (Figs. 2, 3d, and Supplementary Fig. 1). Protein stability using SDM online data server was calculated based on the change in thermal stability (ΔΔG) between the wild type and the new variant. R68 variant was predicted to increase the protein stability while S67, D69 and H159 were detected to decrease protein stability (Table 3).

**Table 3:** Predicted prion protein stability upon mutation in indigenous Afar and Arsi-Bale goat breeds of Ethiopia.

Substitution	WT_ RSA (%)	MT_RSA (%)	WT_OSP	MT_OSP	WT_DEPTH (Å)	MT_DEPTH (Å)	Predicted ΔΔG	Outcome
G67S	0	0	0.93	0.95	8.2	7.3	−0.19	Reduced stability
W68R	13.2	7.5	0.71	0.73	5.5	5.6	0.02	Increased stability
G69D	0	0	0.92	0.94	9	8.1	−0.8	Reduced stability
R159H	40.4	35.4	0.35	0.31	4	3.9	−0.19	Reduced stability
R159H	21.7	13.9	0.42	0.46	4.9	4.8	−1.59	Reduced stability

WT_RSA: wild type relative solvent accessibility in percent; MT_RSA: mutant relative solvent accessibility in percent.; RSA is the absolute value of the net change between the occluded packing density-(OSP) of wild type and mutated residue relative to the average OSP values at solvent exposed regions of the protein. WT_DEPTH (Å): wild type depth in angstrom; MT_DEPTH (Å): mutant depth in angstrom. The stability change by Underlined variant in the above table obtained when PDB −2n53 was used as a template.

Using I-Tasser, residue specific ligand binding probability was predicted for all amino acids in the wild type and variant containing protein sequence. Except R68, the other variants were predicted to have the same probability score. However, the neighbouring residues were predicted to be affected by the substitutions (Supplementary S1).

The MILAMP online database was used to evaluate the amyloid propensity of hot spot heptapeptides in the given sequence with wild type and new variants (Supplementary S1). The wild type sequence GGGWGQP had 0.530 amyloidogenicity score while the same region containing substituted amino acid, GGSRDQP, had 0.029 (Table 4).

**Table 4:** Amyloid forming probability Score of hotspot sequences containing wild type and novel variants of prion protein in two Ethiopian goat breeds.

Start position	Wildtype Sequence	Score	Substituted Sequence	Score
61	GQPHGGG	0.055	GQPHGGS	0.055
62	QPHGGGW	0.055	QPHGGSR	0.053
65	GGGWGQP	0.530	GGSRDQP	0.029
69	GQPHGGG	0.055	DQPHGGG	0.020
64	HGGGWGQ	0.396	HGGSRDQ	0.017
63	PHGGGWG	0.096	PHGGSRD	0.008
66	GGWGQPH	0.154	GSRDQPH	0.005
68	WGQPHGG	0.125	RDQPHGG	0.004
67	GWGQPHG	0.125	SRDQPHG	0.004
153	YRENMYR	0.090	YRENMYH	0.090
154	RENMYRY	0.013	RENMYHY	0.051
155	ENMYRYP	0.090	ENMYHYP	0.292
156	NMYRYPN	0.040	NMYHYPN	0.146
157	MYRYPNQ	0.040	MYHYPNQ	0.146
158	YRYPNQV	0.040	YHYPNQV	0.148
159	RYPNQVY	0.073	HYPNQVY	0.248
			G67S	−0.640
			W68R	−1.000
			G69D	−0.812
			R159H	0.826

Mutation score ([−1, 0) for decreased amyloidogenicity and (0,1] for increased amyloidogenicity.

## Discussion

More than 50 polymorphisms in the goat prion protein-coding gene were reported worldwide^25^. A recent study on Ethiopian goat population reported six variants in Western Highland, Central Highland, and Long Eared Somali breeds of Ethiopia Table 5 ^12^.

**Table 5:** Genotype distribution comparison of polymorphisms in Ethıopian indigenous breeds.

Amino acid Variants	Codon	Present Study			Vitale *et al*. 2019		
		Afar N = 35	Arsi-Bale N = 39	P-Value CI = 95%	Western Highland	Central Highland	Long Eared Somali
67G	GGC	0.51	0.9	0.903			
G67G	GGC–>GGT	0.43	0.02				
G67S	GGC–>AGU	0.06	0.08				
68 W	TGG	0.2	0.9	0.019*			
W68R	TGG–>AGG	0.8	0.1				
69 G	GGC	0.91	0.92	0.884			
G69D	GGC–>GAC	0.09	0.08				
G127	GGC	0.97	0.92	0.837	0.92	0.96	0.97
G127A	GGC–>GCC	0.03	0.08		0.08	0.04	0.03
S138	AGT	0.71	0.69	0.000*			
S138S	AGT–>AGC	0.29	0.31				
H143	CAT	0.97	0.97	0.934	1	1	0.99
H143R	CAT–>CGT	0.03	0.03		—	—	0.01
N146	AAT	0.4	0.46	0.751	0.34	0.44	0.32
N146S	AAT–>AGT	0.6	0.54		0.5	0.42	0.55
R154	CGT	0.86	1	0.789	0.99	1	0.94
R154H	CGT–>CAT	0.14	—		0.091	—	0.05
R159	CGT	0.94	1	0.933			
R159H	CGT–>CAT	0.06	—				
T193	ACC	0.94	0.87	0.049*	0.85	0.86	0.92
T193I	ACC–>ATC	0.06	0.13		0.14	0.13	0.07
Q222	CAG	1	1				
S240	TCC	0.03	0.05	0.251	0.03	0.01	0.02
S240P	TCC–>CCC	0.97	0.95		0.78	0.55	0.78

Silent substitution at codon S138 was observed in 29% of Afar, 31% of Arsi-Bale(present study) and 15% of Tanzanian goat breeds^23^. The frequency of the variant H143R in this study was higher than the previously reported frequency in other goat breeds of Ethiopia as shown in Table 5^12^. Interestingly, the N146S variant seems prevalent in East African goat breeds. In the present study and previously reported Ethiopian breeds, this variant was observed in more than 50% of the study population (Table 4). This variant was also reported in <45 of Tanzanian breeds^23^. The same variant, N146S, has been described in <6% of Anatolian Black goats from Aegean, Mediterranean and South-eastern regions of Turkey^26^. NS146 was reported in 20.4% of healthy Cyproytic goats^27^. R154H variant was detected at a low frequency in Afar(present study), Western highland, Long Eared Somali, and Aegean region goat breeds^12,26^. R154H variant in the Algerian breed has been reported in 15–26% of the study population^9^. The variant T193I was first reported in a recent study by Vitale M. *et al*., 2019 at a frequency similar to the current observation^12^. The overall frequency of serine to proline substitution at 240 positions in the present study was similar to Anatolian goat breeds (0.08)^26^ but higher than the other Ethiopian goat as shown in the above table. G127H143N146R154T193S240 and G127H143S146R154T193P240 were the highest (0.40) haplotypes in a study by Vitale *et al*., 2019. These haplotypes were observed at frequencies of 0.41 and 0.19 in the present study respectively (Supplementary S1).

Previous studies revealed codons which are associated to scrapie susceptibility. The M142 variant which wasn’t identified in the present study was found to delay incubation period^28^. Variants such as S146, D146 and K222 were identified as protective genotypes both in natural outbreaks and experimental challenges^8^. Experimental studies especially supported the potential protective effect of the genotype SS146 by increasing survival rate^29^. As a result, S146K222 haplotype was recommended when crossbreeding by European Food Safety and Authority^8^. In the current study, the S146 variant was prevalent in both breeds compared to the wild type. H143R154 haplotype was observed at high frequency both in Afar and Arsi-Bale breeds. In the study by Billinis *et al*., 2002, this haplotype was prevalent in the majority of scrapie infected animals^30^. In the present study, the wild type R211 and Q222 were detected in all of the population under study. The wild type substitutes at these positions i.e. Q211 and K222 were reported to be protective both in experimental and epidemiological studies^17,31,32^.

In the population under study, the haplotype Q168P240 was highly prevalent. This genotype was detected in scrapie-infected goats in previous studies^30,33^. In an experimental study, animals that carried Q168Q-P240P genotype died of the infection after a prolonged incubation period^34^. A recent work by Langeveld Pirisinu *et al*. 2019 summarized goat scrapie forms and the respective genotypes implicated in each form from different study cases. Genotypes of 127GS, 143HR, 211QR, 240PP, and 240PS were classified under two variants of classical scrapie (CS-1 & CS-2) while 145RH and 240PS were under atypical scrapie (AS)^35^. Accordingly, due to the absence or low frequency of the majority of resistant genotypes, the breeds under study were less resitant to both classical and atypical scrapie.

In this study, we essayed a new perspective on the effect of polymorphisms in goat prion protein stability computationally. Prion protein stability and the process of prion disease development have been linked to the structural alternation of prion protein to potentially amyloid forms^36,37^.

The variation in amino acid sequence found to determine the propensity of amyloid formation^38,39^. In the present study, three consecutive novel polymorphic sites were identified in the first octapeptide repeat of the goat prion gene. Though it is highly conserved, studies confirmed that insertion or deletion in octapeptide repeat has a direct effect on the structural dynamics of the prion protein which in effect could involve in the conversion of cellular prion to scrapie form^20^. A study showed that functional alternation upon mutation triggers conformational flexibility that may cause PrP^Sc^ formation, propagation and disease phenotype induction^39^. Similarly, a study concluded that octapeptide repeats in the human prion protein participate in amyloid formation^40^. In the present study, variant induced secondary and tertiary structure variation of goat prion protein was predicted. Such type of variants were reported to influence the conformational flexibility of ovine PrP and correlated with susceptibility to aggregation in causing different isoforms of scrapie^41,42^. Hence, the investigation of stability and amyloid propensity upon mutation is relevant.

*Insilico* methods may not be as powerful as experimental approaches. However, combining several data sources may increase the validity of the analysis. Here, the prion protein stability and amyloid propensity upon mutation in two Ethiopian goat breeds was predicted using bioinformatic tools. In the predicted model of prion protein (present study), structural variations were observed following the substitutions of the wild type amino acids with new variants as shown in Figs. 2, 3b, and Supplementary Fig. 1. This could be partly due to the difference in the physiochemical properties of the wild types and the substituted variants (Fig. 3a,c). Serine (a derivative of glycine in a reaction that involves two molecules of glycine^43^ at codon 67 was predicted to have a destabilizing effect. This could be because serine is larger in size than glycine (Fig. 3c) and that may create unfavourable torsion angle and bumps in the region. Similarly, there is solubility, hydrophobicity, charge and size difference between tryptophan and arginine. These may influence stability difference at position 68. The substitution of arginine by less soluble and charged aspartic acid at position 69 could be the source of conformational changes in the residue. Since aspartate is a negatively charged molecule, there might be the repulsion of molecules with the same charge and eventually instability in the region^44^.

Though the primary function of prion is not yet clear, it has been implicated in a number of physiological processes like oxidative stress, neural development and metal homeostasis^45,46^. Earlier experimental study reported the link between copper-binding site and prion disease. In a mouse modelled study, the level of copper was the determining factor in prion disease development^47^. In the present study, the residue specific ligand binding probability of R68 (0.026) was predicted to be lower than W68(0.038). The other novel variants rather, seem to have a neutral effect. Amino acids at position 67, 68 and 69 are not in contact with a metal. However, the neighbouring residues i.e. codon 65, 66,72,74,82, 81, 82, 88, 90 and 91 do make a metal-contact as it is annotated in UniProtKB - P52113. H80, G81, G83 and H88 were predicted to be affected by the substitution of the new variants for the wild type (Supplementary Table 3). Therefore, PrP^C^-metal complex formation in the vicinity may be affected by the new variants and lead to a loss or a gain of function^48^.

The N-terminal domain, especially the octapeptide repeat motifs maintain the stability of the globular domain (GD). Physiologically unfavourable interaction of this domain with the surrounding molecules could destabilize the protein^48^. In the present study, the variant H159 was detected in GD region and predicted to decrease stability. The predicted stability effect by H159 was found to be the same when modelled and experimentally derived solution structures (PDB-2n53) were used as a template in the insilico analysis. In this position, arginine is mutated into histidine (smaller in size as indicated in Fig. 3c). The Residue specific ligand binding probabilities of R159 and H159 were predicted to be identical. However, neighbouring residues were seemed to be affected by the substitution. For example, the ligand binding probabilities of W165 in wild type and mutant sequence were predicted to be 0.038 and 0.063 respectively (Supplementary S1). Substitution like this may disturb the ionic interaction and might cause loss of interaction with other molecule such as GRB2, ERI3 and SYN1 as annotated in UniProt- P52113. Similarily, the study by Vitale *et al*. 2019, associated the importance of novel variants in the normal cellular function of prion protein especially its interaction with neural cell adhesion molecules (NCAM)^12^.

Spontaneous misfolding of intrinsically unstable sequence residues may be the underlined cause for sporadic prion diseases. By extrapolating the conformational flexibility of rPrP, a study by Hong Zhang *et al*., 1997, suggested PrP sequence is intrinsically plastic and some domains may tend to favour PrP^c^ to PrP^Sc^ conversion^49^.In the present study, the new variants S67, R68 and D69 were predicted to decrease amyloidogenicity propensity while H159 increased the probability of amyloid formation. The sequence with S67, R68 and D69 i.e. PHGGSRD (0.008) was predicted to have lower amyloidogenicity propensity than the wild type sequence PHGGGWG (0.096). As some proportion of amino acid sequence balance intrinsic amyloid formation and unwanted nucleation, these variants may create favourable conditions in the avoidance of non-physiologic folding^36,38^.Hotspot sequences with the H159 variant exhibited higher amyloidogenicity propensity than the wild type. The relatively higher destabilizing effect of H159 could be the potential reason why heptapeptides that included H159 had higher amyloidogenicity than other novel variants. Hence, this variant could be as important as G127A in the process of amyloid fibril formation^12,50^.

## Conclusion

In the current study, the major resistant variants such as M142, D145, K211, and K222 were not detected. However, other resistant genotypes i.e. H154 in 14% of Afar breed, S146 in >50% and S240 in 8% of the population under study were detected. The present findings indicated the genetic diversity of Ethiopian goat breeds. This study also added valuable information in to the PRNP genetic structure of Ethiopian goat breeds genetic resources and to the world at large. Among the new variants only R68 was found to increase stability. The new variants in the octapeptide repeat domain were predicted to decrease amyloid forming propensity. The variant H159 was predicted to increase amylogenic propensity and reduce stability. These variants could potentially contribute to conformational flexibility of the protein that may trigger the gain or loss of function. Further experimental studies are required to confirm the effect of sequence variation on the structure and function of the prion protein.

## Methods

### Animal selection

In this study, whole blood was collected in EDTA treated tube from jugular veins of 74 unrelated female goats from Afar (n = 35) and from Arsi-Bale (n = 39) breeds. Afar breed is widely distributed in Afar region of Ethiopia. They are adapted to arid area. They are characterized by concave facial profile, narrow faced, forward pointed ears and long horn. They are maintained for milk, meat, and skin production. Arsi- Bale breed is distributed in Arsi, Bale and Western Hararge zones of Oromia region. They are characterized by straight facial profile, backward curved horn, and long ears. They are also reared for meat, milk and skin production (Ethiopian Biodiversity Institute Booklet, 2018).

The sources of the animals for this study were regional research centers (Melka-Were agricultural research center and Adami Tullu Agricultural Research Center). Sample collection and genetic material export permit was assured from Amhara National regional state North Shoa Zone livestock development and promotion office in a letter written on 02/09/2018 and Ethiopian Biodiversity Institute Ref. No. EBI71/943/2018. Animals were treated with a great care and all sampling procedures were according to the institutional guideline.

### DNA extraction and polymerase chain reaction

Genomic DNA was isolated from whole blood samples by using Geneaid DNA isolation kit. A 25 µl final volume PCR reaction of 12.5 µl DreamTaq, PCR Master Mix 2X containing Taq DNA polymerase, dNTPs, MgCl2, and 8.5 µl Nuclease-Free Water(Thermo Scientific), primers; F = 5′-AAAGCCA  CATAGGCAGTT-3′, R = 5′AATGAGGAAAGAGATGAGGAG-3′ (HM038414.1) and 2 µl template DNA was used to amplify the goat prion gene coding region. PCR reaction was carried out with the initial denaturation at 95 °C for 5 min, 36 cycles of denaturation at 94 °C for 45 sec, primer annealing at 58 °C for 45 sec, extension at 72 °C for 45 sec, and final extension at 72 °C for 7 min.

### Sequencing and bioinformatics

1 U Exo-SAP and BigDye™ terminator v3.1 Cycle Sequencing Kit (Thermo Fisher Scientific Inc. USA) was used for incubation and chain termination reaction respectively. Applied Biosystems 3500 genetic analyser (Thermo Fisher Scientific Inc USA) was used for sequencing. FinchTV (http,//www.geospiza.com) was used to visualize the Chromotograms and sequence alignment was performed using Mega v7.0^51^, and Chi-square was executed to compute the Hardy Weinberg Equilibrium using Popgene32^52^. Haplotypes were constructed using phase v2.1 algorithm. Haplotypes with a more than 0.9 post probability score was considered for selecting major haplotypes^53^. Due to some rare genotypes, 50% accuracy was considered to predict haplotypes. Secondary structure was predicted using The MINNUS server-POLYVIEW-2D^54^. Due to the absence of crystallized or solution structure of the first 91 amino acid goat PrP sequence, 3D structure was predicted using I-Tasser online server. Residue specific ligand binding probability and secondary structure is also predicted using the same program. The wild type sequence retrieved from UniProt- P52113 was used as an input amino acid sequence file. The highest C-score model was considered for further stability analysis^55^. A predicted tertiary structure PDB file was used as input in SDM online tool to predict variant effect on stability and relative solvent accessibility^56^. SDM was used to predict stability as it is the least biased pipeline^57^. 3D visualization was obtained using chimera 1.13.1 software package^58^. An experimental goat prion protein solution structure (PDB-2n53) was also used as a template to crosscheck the validity of predicted structure and stability result. Amyloidogenicity propensity of sequences was predicted using Multiple Instance Learning based Amyloid Prediction (MILAMP)^59^. To increase power, 0.941 probability score was considered.

### Ethical approval

Animals were treated with great care and sample was taken according to the institute guideline. Ethical approval is deemed unnecessary according to the Ethiopian National Research Ethics Review Guideline/EFDRE ministry of science and Technology Sep 2014 5th ed. Article 8.3.5.1, 10.2 and 10.5.1. Consent was granted to take blood samples from the respective regional state livestock development and promotion offices NS/AR/U-01/42/2010 and NS/AR/U-01/41/2010. Genetic material export permit was assured from Ethiopian Biodiversity Institute, Ref. No. EBI71/943/2018.

## Supplementary information

Supplementary Figure 1.

Supplementary Information.

## Data Availability

The datasets generated during and/or analysed during the current study are available in the Genbank MN795374-MN795447.
