# Supplementary material for: Novel Variations in Native Ethiopian Goat breeds PRNP Gene and Their Potential Effect on Prion Protein Stability
Source: Sci Rep. 2020 Apr 24;10:6953. doi: 10.1038/s41598-020-63874-z (PMC7181617; doi:10.1038/s41598-020-63874-z)
Supplement: Supplementary file 1 — Supplementary Figure 1. [file 41598_2020_63874_MOESM1_ESM.docx]

Novel Variations in Native Ethiopian Goat breeds Prnp Gene and Their Potential Effect on Prion Protein Stability

**Eden Yitna Teferedegn^1^ , Yalçın Yaman^2^ and Cemal Ün^1^***

^1^Ege University, Department of Biology, Molecular Biology Division, Izmir, Turkey.

^2^Department of Biometry and Genetics, Bandırma Sheep Research Institute, Bandırma, Balıkesir, Turkey.

*[cemaluen@gmail.com](mailto:cemaluen@gmail.com)


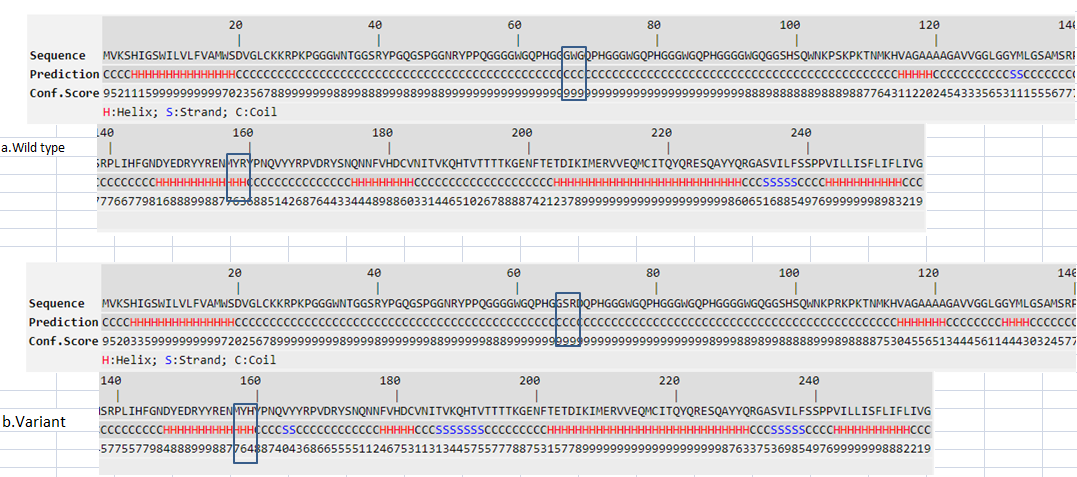


Sup. Figure 1 Predicted secondar structure of the wild type and varint.Boxes indicate the postion of substitutions.H: Helix;S: Strand and C: coil.a. wild type, b.variant.
