# Supplementary material for: Novel Variations in Native Ethiopian Goat breeds PRNP Gene and Their Potential Effect on Prion Protein Stability
Source: Sci Rep. 2020 Apr 24;10:6953. doi: 10.1038/s41598-020-63874-z (PMC7181617; doi:10.1038/s41598-020-63874-z)
Supplement: Supplementary file 2 — Supplementary Information. [file 41598_2020_63874_MOESM2_ESM.pdf]

Novel Variations in Native Ethiopian Goat breeds Prnp Gene and Their Potential Effect on Prion Protein Stability

Eden Yitna Teferedegn[1] , Yalcin Yaman[2] and Cemal Un1*

Table 1 Haplotype

		HAPLOTYPE 1						
		67NOV	68NOV	69NOV	127	138	143	146
AfCap01		G	W	G	G	S	H	N
AfCap02		G	R	G	G	S	H	S
AfCap03		G	W	G	G	S	H	S
AfCap04		G	W	G	G	S	H	S
AfCap05		G	W	G	G	S	H	S
AfCap06		G	W	G	G	S	H	N
AfCap07		G	R	G	G	S	H	N
AfCap08		G	R	G	G	S	H	S
AfCap09		G	W	G	G	S	H	N
AfCap10		G	W	G	G	S	H	N
AfCap11		G	R	G	G	S	H	N
AfCap12		G	W	G	G	S	H	S
AfCap13		G	W	G	G	S	H	N
AfCap14		G	W	G	G	S	H	S
AfCap15		G	W	G	G	S	H	N
AfCap16		G	W	G	G	S	H	S
AfCap17		G	W	G	G	S	H	S
AfCap18		G	W	G	G	S	H	N
AfCap19		G	W	G	G	S	H	N
AfCap20		G	R	G	A	S	H	N
AfCap23		G	W	G	G	S	H	S
AfCap24		G	W	G	G	S	H	S
AfCap25		S	R	G	G	S	H	N
AfCap26		S	R	G	G	S	H	S
AfCap27		G	W	G	G	S	H	S
AfCap28		G	W	G	G	S	H	S
AfCap29		G	W	G	G	S	H	N
AfCap30		G	W	G	G	S	H	S
AfCap31		G	W	G	G	S	H	S
AfCap35		G	W	G	G	S	H	N
AfCap36		G	W	G	G	S	H	S
AfCap37		G	W	G	G	S	H	N
AfCap38		G	R	G	G	S	H	N
AfCap39		G	W	G	G	S	H	S
AfCap40		G	W	G	G	S	H	N
ArCap01		S	R	D	G	S	H	N
ArCap02		S	R	D	G	S	H	N
ArCap03		G	W	G	G	S	H	S
ArCap05		S	R	D	A	S	H	N
ArCap06		G	W	G	G	S	H	N

ArCap07	?	G	W	G	G	S	H	S
ArCap08	?	G	W	G	G	S	H	S
ArCap09	?	G	W	G	G	S	H	S
ArCap10	?	G	W	G	G	S	H	N
ArCap11	?	G	W	G	G	S	H	N
ArCap12	?	G	W	G	G	S	H	N
ArCap13	?	G	W	G	G	S	H	N
ArCap14	?	G	W	G	G	S	H	S
ArCap15	?	G	W	G	G	S	H	S
ArCap16	?	G	W	G	G	S	H	S
ArCap17	?	G	W	G	G	S	H	S
ArCap18	?	G	W	G	G	S	H	S
ArCap19	?	G	W	G	G	S	H	S
ArCap20	?	G	W	G	G	S	H	N
ArCap21	?	G	W	G	G	S	H	S
ArCap22	?	G	W	G	G	S	H	N
ArCap23	?	G	W	G	G	S	H	S
ArCap24	?	G	W	G	G	S	H	N
ArCap25	?	G	W	G	G	S	H	N
ArCap26	?	G	W	G	G	S	H	N
ArCap27	?	G	W	G	G	S	H	N
ArCap28	?	G	W	G	G	S	H	N
ArCap29	?	G	W	G	G	S	H	S
ArCap30	?	G	W	G	G	S	H	S
ArCap31	?	G	W	G	G	S	H	N
ArCap32	?	G	W	G	G	S	H	N
ArCap33	?	G	W	G	G	S	H	N
ArCap34	?	G	W	G	G	S	H	N
ArCap35	?	G	W	G	G	S	H	N
ArCap36	?	G	W	G	G	S	H	N
ArCap37	?	G	W	G	G	S	H	S
ArCap38	?	G	W	G	G	S	H	N
ArCap39	?	G	W	G	G	S	H	S
ArCap40	?	G	W	G	G	S	H	N

AfCap-Afar Caprine, ArCap-Arsi Caprine

*Haplotype Blockf from the Each partent (HAPLOTYPE 1 ve 2)

* *Colured columns are MONOMORPHIC lucus

***Last column best prediction

154	159NOV	193	240		67NOV	68NOV	69NOV	127
R	R	T	P		G	R	G	G
R	R	T	P		G	W	G	G
R	R	T	P		G	R	G	G
R	R	T	P		G	R	G	G
R	R	T	P		G	R	G	G
R	R	T	P		G	R	G	G
R	R	T	P		G	W	G	G
R	R	T	P		G	W	G	G
R	R	T	S		G	W	G	G
R	R	T	P		G	W	G	G
R	R	T	P		G	W	G	G
R	R	T	P		G	R	G	G
R	R	T	P		G	R	G	G
R	R	T	P		G	W	G	G
R	R	I	P		G	R	G	G
R	R	T	P		G	R	G	G
R	R	T	P		G	R	G	G
R	R	T	P		G	R	G	G
R	R	T	P		G	R	G	G
R	R	T	P		G	W	G	G
R	R	T	P		G	R	G	G
R	R	T	P		G	R	G	G
H	R	T	P		G	W	G	G
R	R	T	P		G	W	G	G
R	R	T	P		G	R	G	G
R	R	T	P		G	R	G	G
R	R	T	P		G	R	G	G
R	R	T	P		G	R	G	G
R	R	T	P		G	R	G	G
R	R	T	P		G	W	G	G
R	R	T	P		G	R	G	G
R	R	T	P		G	W	G	G
R	R	T	P		G	W	G	G
R	R	T	P		G	W	G	G
R	R	T	P		G	W	G	G
R	R	T	P		G	W	G	G
R	R	T	P		G	W	G	G
R	R	T	P		G	W	G	G
R	R	T	P		G	W	G	G
R	R	T	P		G	W	G	G

R	R	T	P		G	W	G	G
R	R	T	P		G	W	G	G
R	R	T	P		G	W	G	G
R	R	T	P		G	W	G	G
R	R	T	S		G	W	G	G
R	R	T	P		G	W	G	A
R	R	T	P		G	W	G	G
R	R	T	P		G	W	G	G
R	R	T	P		G	W	G	G
R	R	T	P		G	W	G	G
R	R	T	P		G	W	G	G
R	R	T	P		G	W	G	G
R	R	T	P		G	W	G	G
R	R	T	P		G	W	G	G
R	R	T	P		G	W	G	G
R	R	T	P		G	W	G	G
R	R	T	P		G	W	G	G
R	R	T	P		G	W	G	G
R	R	T	P		G	W	G	G
R	R	T	P		G	W	G	G
R	R	T	P		G	W	G	G
R	R	T	P		G	W	G	G
R	R	T	P		G	R	G	G
R	R	T	P		G	W	G	G
R	R	T	P		G	W	G	G
R	R	T	P		G	W	G	G
R	R	T	S		G	W	G	G
R	R	T	P		G	W	G	G
R	R	T	P		G	W	G	G
R	R	T	P		G	W	G	G
R	R	T	P		G	W	G	G
R	R	I	P		G	W	G	G
R	R	T	P		G	W	G	G
R	R	T	P		G	W	G	A

HAPLOTYPE 2

138	143	146	154	159NOV	193	240
S	H	S	R	H	T	P
S	H	N	R	R	T	S
S	H	S	R	R	T	S
S	H	N	R	R	I	P
S	H	N	R	R	T	P
S	H	N	R	R	T	P
S	H	N	R	R	T	S
S	H	N	H	R	T	S
S	H	N	H	R	T	S
S	H	N	R	R	T	S
S	H	N	R	R	T	S
S	H	N	R	R	T	P
S	H	N	R	R	T	P
S	H	N	H	R	T	P
S	H	N	R	R	I	P
S	H	N	R	R	T	P
S	H	S	R	R	T	P
S	H	N	R	R	T	P
S	H	N	R	R	T	P
S	H	S	R	R	T	P
S	H	N	R	R	T	P
S	H	N	R	R	T	P
S	H	N	R	R	T	P
S	H	S	R	R	T	P
S	H	N	R	R	T	P
S	H	N	R	R	T	P
S	H	S	R	H	T	P
S	H	N	R	R	T	P
S	H	N	R	R	T	P
S	H	N	H	R	T	P
S	H	N	R	R	T	P
S	R	N	R	R	T	P
S	H	N	R	R	T	S
S	H	N	R	R	T	P
S	H	N	R	R	T	S
S	H	N	R	R	T	P
S	H	S	R	R	T	P
S	H	S	R	R	T	P
S	H	N	R	R	T	P
S	H	S	R	R	T	P

S	H	S	R	R	T	P
S	H	N	R	R	T	S
S	H	N	R	R	I	P
S	H	N	R	R	T	S
S	H	N	R	R	T	S
S	H	N	R	R	T	P
S	H	S	R	R	T	P
S	H	S	R	R	T	P
S	H	S	R	R	T	P
S	H	N	R	R	I	P
S	H	N	R	R	T	S
S	H	S	R	R	T	P
S	H	N	R	R	T	S
S	H	N	R	R	T	P
S	H	N	R	R	T	S
S	H	S	R	R	T	P
S	H	N	R	R	T	S
S	H	S	R	R	T	P
S	H	N	R	R	T	S
S	H	N	R	R	T	P
S	H	N	R	R	T	P
S	R	N	R	R	T	P
S	H	N	R	R	T	P
S	H	S	R	R	T	P
S	H	S	R	R	T	P
S	H	N	R	R	T	S
S	H	N	R	R	T	S
S	H	N	R	R	T	P
S	H	N	R	R	T	P
S	H	N	R	R	I	P
S	H	N	R	R	I	P
S	H	N	R	R	T	S
S	H	S	R	R	T	P
S	H	N	R	R	T	P

,

HAPLOTYPE 1	HAPLOTYPE 2	POSTPROBABILITY
GWGGSHNRRTP	GRGGSHSRHTP	1
GRGGSHSRRTP	GWGGSHNRRTS	1
GWGGSHSRRTP	GRGGSHSRRTS	1
GWGGSHSRRTP	GRGGSHNRRIP	0.5914
GWGGSHSRRTP	GRGGSHNRRTP	0.6887
GWGGSHNRRTP	GRGGSHNRRTP	1
GRGGSHNRRTP	GWGGSHNRRTS	1
GRGGSHSRRTP	GWGGSHNHRTS	0.9237
GWGGSHNRRTS	GWGGSHNHRTS	0.5
GWGGSHNRRTP	GWGGSHNRRTS	1
GRGGSHNRRTP	GWGGSHNRRTS	1
GWGGSHSRRTP	GRGGSHNRRTP	0.6887
GWGGSHNRRTP	GRGGSHNRRTP	1
GWGGSHSRRTP	GWGGSHNHRTP	0.5
GWGGSHNRRIP	GRGGSHNRRIP	1
GWGGSHSRRTP	GRGGSHNRRTP	0.6887
GWGGSHSRRTP	GRGGSHSRRTP	1
GWGGSHNRRTP	GRGGSHNRRTP	1
GWGGSHNRRTP	GRGGSHNRRTP	1
GRGASHNRRTP	GWGGSHSRRTP	0.5848
GWGGSHSRRTP	GRGGSHNRRTP	0.6887
GWGGSHSRRTP	GRGGSHNRRTP	0.6887
SRGGSHNHRTP	GWGGSHNRRTP	1
SRGGSHSRRTP	GWGGSHSRRTP	1
GWGGSHSRRTP	GRGGSHNRRTP	0.6887
GWGGSHSRRTP	GRGGSHNRRTP	0.6887
GWGGSHNRRTP	GRGGSHSRHTP	1
GWGGSHSRRTP	GRGGSHNRRTP	0.6887
GWGGSHSRRTP	GRGGSHNRRTP	0.6887
GWGGSHNRRTP	GWGGSHNHRTP	0.9742
GWGGSHSRRTP	GRGGSHNRRTP	0.6887
GWGGSHNRRTP	GWGGSRNRRTP	1
GRGGSHNRRTP	GWGGSHNRRTS	1
GWGGSHSRRTP	GWGGSHNRRTP	1
GWGGSHNRRTP	GWGGSHNRRTS	1
SRDGSHNRRTP	GWGGSHNRRTP	1
SRDGSHNRRTP	GWGGSHSRRTP	1
GWGGSHSRRTP	GWGGSHSRRTP	1
SRDASHNRRTP	GWGGSHNRRTP	1
GWGGSHNRRTP	GWGGSHSRRTP	0.5

GWGGSHSRRTP	GWGGSHSRRTP	1
GWGGSHSRRTP	GWGGSHNRRTS	1
GWGGSHSRRTP	GWGGSHNRRIP	0.5
GWGGSHNRRTP	GWGGSHNRRTS	1
GWGGSHNRRTS	GWGGSHNRRTS	1
GWGGSHNRRTP	GWGASHNRRTP	1
GWGGSHNRRTP	GWGGSHSRRTP	0.5
GWGGSHSRRTP	GWGGSHSRRTP	1
GWGGSHSRRTP	GWGGSHSRRTP	1
GWGGSHSRRTP	GWGGSHNRRIP	0.5
GWGGSHSRRTP	GWGGSHNRRTS	1
GWGGSHSRRTP	GWGGSHSRRTP	1
GWGGSHSRRTP	GWGGSHNRRTS	1
GWGGSHNRRTP	GWGGSHNRRTP	1
GWGGSHSRRTP	GWGGSHNRRTS	1
GWGGSHNRRTP	GWGGSHSRRTP	0.5
GWGGSHSRRTP	GWGGSHNRRTS	1
GWGGSHNRRTP	GWGGSHSRRTP	0.5
GWGGSHNRRTP	GWGGSHNRRTS	1
GWGGSHNRRTP	GWGGSHNRRTP	1
GWGGSHNRRTP	GWGGSHNRRTP	1
GWGGSHNRRTP	GWGGSRNRRTP	1
GWGGSHSRRTP	GRGGSHNRRTP	0.6887
GWGGSHSRRTP	GWGGSHSRRTP	1
GWGGSHNRRTP	GWGGSHSRRTP	0.5
GWGGSHNRRTP	GWGGSHNRRTS	1
GWGGSHNRRTS	GWGGSHNRRTS	1
GWGGSHNRRTP	GWGGSHNRRTP	1
GWGGSHNRRTP	GWGGSHNRRTP	1
GWGGSHNRRTP	GWGGSHNRRIP	0.5
GWGGSHSRRTP	GWGGSHNRRIP	0.5
GWGGSHNRRIP	GWGGSHNRRTS	1
GWGGSHSRRTP	GWGGSHSRRTP	1
GWGGSHNRRTP	GWGASHNRRTP	1

Table 2 Hotspot identification Pro

Mutation score ([-1,0) for decreased amyloidogenicity and (0,1] for increased amyloidogenicity):

Wild type sequecne

Sr#	Startposition	sequence	score
1	10	ILVLFVA	0.941
2	9	WILVLFV	0.934
3	12	VLFVAMW	0.925
4	11	LVLFVAM	0.923
5	250	LIFLIVG	0.919
6	13	LFVAMWS	0.918
7	251	IFLIVG	0.898
8	182	CVNITVK	0.898
9	120	AAGAVVG	0.896
10	14	FVAMWSD	0.893
11	249	FLIFLIV	0.886
12	8	SWILVLF	0.881
13	174	NQNNFVH	0.876
14	233	ASVILFS	0.867
15	243	VILLISF	0.856
16	247	ISFLIFL	0.853
17	121	AGAVVGG	0.852
18	7	GSWILVL	0.851
19	123	AVVGGLG	0.832
20	115	VAGAAAA	0.826
21	119	AAAGAVV	0.826
22	252	FLIVG	0.814
23	235	VILFSSP	0.808
24	234	SVILFSS	0.808
25	6	IGSWILV	0.805
26	246	LISFLIF	0.801
27	244	ILLISFL	0.801
28	122	GAVVGGL	0.793
29	175	QNNFVHD	0.792
30	248	SFLIFLI	0.792
31	232	GASVILF	0.777
32	124	VVGGLGG	0.768
33	212	VVEQMCI	0.729

Novel Variant substituted sequence only at oc

Sr#	Startposition	sequence	score
1	10	ILVLFVA	0.941
2	9	WILVLFV	0.934
3	12	VLFVAMW	0.925
4	11	LVLFVAM	0.923
5	250	LIFLIVG	0.919
6	13	LFVAMWS	0.918
7	251	IFLIVG*	0.898
8	182	CVNITVK	0.898
9	120	AAGAVVG	0.896
10	14	FVAMWSD	0.893
11	249	FLIFLIV	0.886
12	8	SWILVLF	0.881
13	174	NQNNFVH	0.876
14	233	ASVILFS	0.867
15	243	VILLISF	0.856
16	247	ISFLIFL	0.853
17	121	AGAVVGG	0.852
18	7	GSWILVL	0.851
19	123	AVVGGLG	0.832
20	115	VAGAAAA	0.826
21	119	AAAGAVV	0.826
22	252	FLIVG*	0.814
23	235	VILFSSP	0.808
24	234	SVILFSS	0.808
25	6	IGSWILV	0.805
26	246	LISFLIF	0.801
27	244	ILLISFL	0.801
28	122	GAVVGGL	0.793
29	175	QNNFVHD	0.792
30	248	SFLIFLI	0.792
31	232	GASVILF	0.777
32	124	VVGGLGG	0.768

34	245	LLISFLI	0.725
35	116	AGAAAAG	0.721
36	118	AAAAGAV	0.721
37	5	HIGSWIL	0.714
38	173	SNQNNFV	0.714
39	114	HVAGAAA	0.711
40	217	CITQYQR	0.678
41	216	MCITQYQ	0.676
42	178	FVHDCVN	0.665
43	215	QMCITQY	0.661
44	213	VEQMCIT	0.660
45	172	YSNQNNF	0.658
46	225	SQAYYQR	0.650
47	141	LIHFGND	0.647
48	117	GAAAAGA	0.633
49	15	VAMWSDV	0.613
50	16	AMWSDVG	0.613
51	179	VHDCVNI	0.606
52	131	YMLGSAM	0.552
53	4	SHIGSWI	0.550
54	125	VGGLGGY	0.546
55	98	SHSQWNK	0.545
56	132	MLGSAMS	0.532
57	181	DCVNITV	0.531
58	55	QGGGGWG	0.530
59	92	GWGQGGS	0.530
60	91	GGWGQGG	0.530
61	81	GGGWGQP	0.530
62	73	GGGWGQP	0.530
63	65	GGGWGQP	0.530
64	57	GGGWGQP	0.530
65	90	GGGWGQG	0.530
66	180	HDCVNIT	0.526
67	93	WGQGGSH	0.523
68	128	LGGYMLG	0.522
69	17	MWSDVGL	0.513
70	177	NFVHDCV	0.511

33	212	VVEQMCI	0.729
34	245	LLISFLI	0.725
35	116	AGAAAAG	0.721
36	118	AAAAGAV	0.721
37	5	HIGSWIL	0.714
38	173	SNQNNFV	0.714
39	114	HVAGAAA	0.711
40	253	LIVG*	0.698
41	217	CITQYQR	0.678
42	216	MCITQYQ	0.676
43	178	FVHDCVN	0.665
44	215	QMCITQY	0.661
45	213	VEQMCIT	0.660
46	172	YSNQNNF	0.658
47	225	SQAYYQR	0.650
48	141	LIHFGND	0.647
49	117	GAAAAGA	0.633
50	15	VAMWSDV	0.613
51	16	AMWSDVG	0.613
52	179	VHDCVNI	0.606
53	131	YMLGSAM	0.552
54	4	SHIGSWI	0.550
55	125	VGGLGGY	0.546
56	98	SHSQWNK	0.545
57	132	MLGSAMS	0.532
58	181	DCVNITV	0.531
59	55	QGGGGWG	0.530
60	92	GWGQGGS	0.530
61	91	GGWGQGG	0.530
62	81	GGGWGQP	0.530
63	73	GGGWGQP	0.530
64	57	GGGWGQP	0.530
65	90	GGGWGQG	0.530
66	180	HDCVNIT	0.526
67	93	WGQGGSH	0.523
68	128	LGGYMLG	0.522
69	17	MWSDVGL	0.513

71	183	VNITVKQ	0.509
72	18	WSDVGLC	0.506
73	176	NNFVHDC	0.501
74	242	PVILLIS	0.497
75	33	GWNTGGS	0.491
76	32	GGWNTGG	0.491
77	31	GGGWNTG	0.491
78	34	WNTGGSR	0.484
79	127	GLGGYML	0.458
80	129	GGYMLGS	0.458
81	89	GGGGWGQ	0.456
82	56	GGGGWGQ	0.456
83	97	GSHSQWN	0.454
84	133	LGSAMSR	0.453
85	130	GYMLGSA	0.450
86	162	NQVYYRP	0.414
87	80	HGGGWGQ	0.396
88	72	HGGGWGQ	0.396
89	64	HGGGWGQ	0.396
90	88	HGGGGWG	0.396
91	126	GGLGGYM	0.387
92	19	SDVGLCK	0.384
93	160	YPNQVYY	0.378
94	161	PNQVYYR	0.378
95	214	EQMCITQ	0.360
96	224	ESQAYYQ	0.350
97	184	NITVKQH	0.337
98	231	RGASVIL	0.335
99	142	IHFGNDY	0.334
100	189	QHTVTTT	0.332
101	208	IMERVVE	0.330
102	187	VKQHTVT	0.276
103	111	NMKHVAG	0.273
104	143	HFGNDYE	0.267
105	226	QAYYQRG	0.265
106	94	GQGGSHS	0.257
107	230	QRGASVI	0.255

70	177	NFVHDCV	0.511
71	183	VNITVKQ	0.509
72	18	WSDVGLC	0.506
73	176	NNFVHDC	0.501
74	242	PVILLIS	0.497
75	33	GWNTGGS	0.491
76	32	GGWNTGG	0.491
77	31	GGGWNTG	0.491
78	34	WNTGGSR	0.484
79	127	GLGGYML	0.458
80	129	GGYMLGS	0.458
81	89	GGGGWGQ	0.456
82	56	GGGGWGQ	0.456
83	97	GSHSQWN	0.454
84	133	LGSAMSR	0.453
85	130	GYMLGSA	0.450
86	162	NQVYYRP	0.414
87	80	HGGGWGQ	0.396
88	72	HGGGWGQ	0.396
89	88	HGGGGWG	0.396
90	126	GGLGGYM	0.387
91	19	SDVGLCK	0.384
92	160	YPNQVYY	0.378
93	161	PNQVYYR	0.378
94	214	EQMCITQ	0.360
95	224	ESQAYYQ	0.350
96	184	NITVKQH	0.337
97	231	RGASVIL	0.335
98	142	IHFGNDY	0.334
99	189	QHTVTTT	0.332
100	208	IMERVVE	0.330
101	187	VKQHTVT	0.276
102	111	NMKHVAG	0.273
103	143	HFGNDYE	0.267
104	226	QAYYQRG	0.265
105	94	GQGGSHS	0.257
106	230	QRGASVI	0.255

108	95	QGGSHSQ	0.251
109	96	GGSHSQW	0.251
110	211	RVVEQMC	0.251
111	1	MVKSHIG	0.249
112	191	TVTTTTK	0.231
113	190	HTVTTTT	0.227
114	113	KHVAGAA	0.226
115	185	ITVKQHT	0.216
116	228	YYQRGAS	0.211
117	227	AYYQRGA	0.211
118	218	ITQYQRE	0.208
119	112	MKHVAGA	0.206
120	171	RYSNQNN	0.200
121	2	VKSHIGS	0.199
122	236	ILFSSPP	0.198
123	99	HSQWNKP	0.180
124	140	PLIHFGN	0.170
125	110	TNMKHVA	0.166
126	30	PGGGWNT	0.164
127	82	GGWGQPH	0.154
128	74	GGWGQPH	0.154
129	66	GGWGQPH	0.154
130	58	GGWGQPH	0.154
131	144	FGNDYED	0.154
132	229	YQRGASV	0.153
133	164	VYYRPVD	0.139
134	84	WGQPHGG	0.125
135	76	WGQPHGG	0.125
136	68	WGQPHGG	0.125
137	60	WGQPHGG	0.125
138	83	GWGQPHG	0.125
139	75	GWGQPHG	0.125
140	67	GWGQPHG	0.125
141	59	GWGQPHG	0.125
142	169	VDRYSNQ	0.111
143	134	GSAMSRP	0.107
144	20	DVGLCKK	0.103

107	95	QGGSHSQ	0.251
108	96	GGSHSQW	0.251
109	211	RVVEQMC	0.251
110	1	MVKSHIG	0.249
111	191	TVTTTTK	0.231
112	190	HTVTTTT	0.227
113	113	KHVAGAA	0.226
114	185	ITVKQHT	0.216
115	228	YYQRGAS	0.211
116	227	AYYQRGA	0.211
117	218	ITQYQRE	0.208
118	112	MKHVAGA	0.206
119	171	RYSNQNN	0.200
120	2	VKSHIGS	0.199
121	236	ILFSSPP	0.198
122	99	HSQWNKP	0.180
123	140	PLIHFGN	0.170
124	110	TNMKHVA	0.166
125	30	PGGGWNT	0.164
126	82	GGWGQPH	0.154
127	74	GGWGQPH	0.154
128	58	GGWGQPH	0.154
129	144	FGNDYED	0.154
130	229	YQRGASV	0.153
131	164	VYYRPVD	0.139
132	84	WGQPHGG	0.125
133	76	WGQPHGG	0.125
134	60	WGQPHGG	0.125
135	83	GWGQPHG	0.125
136	75	GWGQPHG	0.125
137	59	GWGQPHG	0.125
138	169	VDRYSNQ	0.111
139	134	GSAMSRP	0.107
140	20	DVGLCKK	0.103
141	186	TVKQHTV	0.100
142	188	KQHTVTT	0.100
143	198	GENFTET	0.099

145	186	TVKQHTV	0.100
146	188	KQHTVTT	0.100
147	198	GENFTET	0.099
148	201	FTETDIK	0.099
149	79	PHGGGWG	0.096
150	71	PHGGGWG	0.096
151	63	PHGGGWG	0.096
152	41	YPGQGSP	0.094
153	152	YYRENMY	0.090
154	153	YRENMYR	0.090
155	155	ENMYRYP	0.090
156	210	ERVVEQM	0.089
157	209	MERVVEQ	0.088
158	204	TDIKIME	0.082
159	199	ENFTETD	0.081
160	3	KSHIGSW	0.081
161	241	PPVILLI	0.075
162	200	NFTETDI	0.074
163	159	RYPNQVY	0.073
164	163	QVYYRPV	0.073
165	45	GSPGGNR	0.071
166	54	PQGGGGW	0.069
167	44	QGSPGGN	0.067
168	43	GQGSPGG	0.067
169	35	NTGGSRY	0.066
170	36	TGGSRYP	0.066
171	223	RESQAYY	0.065
172	192	VTTTTKG	0.063
173	222	QRESQAY	0.061
174	221	YQRESQA	0.059
175	220	QYQRESQ	0.059
176	170	DRYSNQN	0.058
177	240	SPPVILL	0.057
178	78	QPHGGGW	0.055
179	70	QPHGGGW	0.055
180	62	QPHGGGW	0.055
181	86	QPHGGGG	0.055

144	201	FTETDIK	0.099
145	79	PHGGGWG	0.096
146	71	PHGGGWG	0.096
147	41	YPGQGSP	0.094
148	152	YYRENMY	0.090
149	153	YRENMYR	0.090
150	155	ENMYRYP	0.090
151	210	ERVVEQM	0.089
152	209	MERVVEQ	0.088
153	204	TDIKIME	0.082
154	199	ENFTETD	0.081
155	3	KSHIGSW	0.081
156	241	PPVILLI	0.075
157	200	NFTETDI	0.074
158	159	RYPNQVY	0.073
159	163	QVYYRPV	0.073
160	45	GSPGGNR	0.071
161	54	PQGGGGW	0.069
162	44	QGSPGGN	0.067
163	43	GQGSPGG	0.067
164	35	NTGGSRY	0.066
165	36	TGGSRYP	0.066
166	223	RESQAYY	0.065
167	192	VTTTTKG	0.063
168	222	QRESQAY	0.061
169	221	YQRESQA	0.059
170	220	QYQRESQ	0.059
171	170	DRYSNQN	0.058
172	240	SPPVILL	0.057
173	78	QPHGGGW	0.055
174	70	QPHGGGW	0.055
175	86	QPHGGGG	0.055
176	61	GQPHGGS	0.055
177	85	GQPHGGG	0.055
178	77	GQPHGGG	0.055
179	21	VGLCKKR	0.054
180	62	QPHGGSR	0.053

182	85	GQPHGGG	0.055
183	77	GQPHGGG	0.055
184	69	GQPHGGG	0.055
185	61	GQPHGGG	0.055
186	21	VGLCKKR	0.054
187	219	TQYQRES	0.049
188	196	TKGENFT	0.048
189	197	KGENFTE	0.048
190	100	SQWNKPR	0.044
191	239	SSPPVIL	0.044
192	205	DIKIMER	0.042
193	87	PHGGGGW	0.041
194	238	FSSPPVI	0.041
195	158	YRYPNQV	0.040
196	156	NMYRYPN	0.040
197	157	MYRYPNQ	0.040
198	139	RPLIHFG	0.037
199	145	GNDYEDR	0.033
200	137	MSRPLIH	0.033
201	207	KIMERVV	0.031
202	148	YEDRYYR	0.026
203	203	ETDIKIM	0.025
204	136	AMSRPLI	0.025
205	193	TTTTKGE	0.024
206	29	KPGGGWN	0.024
207	206	IKIMERV	0.023
208	195	TTKGENF	0.021
209	47	PGGNRYP	0.021
210	48	GGNRYPP	0.021
211	165	YYRPVDR	0.020
212	237	LFSSPPV	0.020
213	138	SRPLIHF	0.020
214	40	RYPGQGS	0.020
215	135	SAMSRPL	0.019
216	39	SRYPGQG	0.019
217	52	YPPQGGG	0.017
218	46	SPGGNRY	0.014

181	219	TQYQRES	0.049
182	196	TKGENFT	0.048
183	197	KGENFTE	0.048
184	100	SQWNKPR	0.044
185	239	SSPPVIL	0.044
186	205	DIKIMER	0.042
187	87	PHGGGGW	0.041
188	238	FSSPPVI	0.041
189	158	YRYPNQV	0.040
190	156	NMYRYPN	0.040
191	157	MYRYPNQ	0.040
192	139	RPLIHFG	0.037
193	145	GNDYEDR	0.033
194	137	MSRPLIH	0.033
195	207	KIMERVV	0.031
196	65	GGSRDQP	0.029
197	148	YEDRYYR	0.026
198	203	ETDIKIM	0.025
199	136	AMSRPLI	0.025
200	193	TTTTKGE	0.024
201	29	KPGGGWN	0.024
202	206	IKIMERV	0.023
203	195	TTKGENF	0.021
204	47	PGGNRYP	0.021
205	48	GGNRYPP	0.021
206	165	YYRPVDR	0.020
207	69	DQPHGGG	0.020
208	237	LFSSPPV	0.020
209	138	SRPLIHF	0.020
210	40	RYPGQGS	0.020
211	135	SAMSRPL	0.019
212	39	SRYPGQG	0.019
213	52	YPPQGGG	0.017
214	64	HGGSRDQ	0.017
215	46	SPGGNRY	0.014
216	38	GSRYPGQ	0.014
217	37	GGSRYPG	0.014

219	38	GSRYPGQ	0.014
220	37	GGSRYPG	0.014
221	168	PVDRYSN	0.014
222	151	RYYRENM	0.014
223	154	RENMYRY	0.013
224	109	KTNMKHV	0.013
225	53	PPQGGGG	0.012
226	194	TTTKGEN	0.012
227	42	PGQGSPG	0.011
228	202	TETDIKI	0.010
229	101	QWNKPRK	0.009
230	146	NDYEDRY	0.006
231	147	DYEDRYY	0.006
232	50	NRYPPQG	0.005
233	22	GLCKKRP	0.004
234	49	GNRYPPQ	0.003
235	149	EDRYYRE	0.003
236	150	DRYYREN	0.003
237	51	RYPPQGG	0.003
238	166	YRPVDRY	0.003
239	167	RPVDRYS	0.003
240	108	PKTNMKH	0.003
241	107	KPKTNMK	0.003
242	28	PKPGGGW	0.002
243	102	WNKPRKP	0.001
244	23	LCKKRPK	0.001
245	106	RKPKTNM	0.000
246	27	RPKPGGG	0.000
247	24	CKKRPKP	0.000
248	103	NKPRKPK	0.000
249	26	KRPKPGG	0.000
250	105	PRKPKTN	0.000
251	104	KPRKPKT	0.000
252	25	KKRPKPG	0.000

218	168	PVDRYSN	0.014
219	151	RYYRENM	0.014
220	154	RENMYRY	0.013
221	109	KTNMKHV	0.013
222	53	PPQGGGG	0.012
223	194	TTTKGEN	0.012
224	42	PGQGSPG	0.011
225	202	TETDIKI	0.010
226	101	QWNKPRK	0.009
227	63	PHGGSRD	0.008
228	146	NDYEDRY	0.006
229	147	DYEDRYY	0.006
230	66	GSRDQPH	0.005
231	50	NRYPPQG	0.005
232	22	GLCKKRP	0.004
233	68	RDQPHGG	0.004
234	67	SRDQPHG	0.004
235	49	GNRYPPQ	0.003
236	149	EDRYYRE	0.003
237	150	DRYYREN	0.003
238	51	RYPPQGG	0.003
239	166	YRPVDRY	0.003
240	167	RPVDRYS	0.003
241	108	PKTNMKH	0.003
242	107	KPKTNMK	0.003
243	28	PKPGGGW	0.002
244	102	WNKPRKP	0.001
245	23	LCKKRPK	0.001
246	106	RKPKTNM	0.000
247	27	RPKPGGG	0.000
248	24	CKKRPKP	0.000
249	103	NKPRKPK	0.000
250	26	KRPKPGG	0.000
251	105	PRKPKTN	0.000
252	104	KPRKPKT	0.000

bability Score

ctareapt	Novel Variant substituted sequence -H159		
1	10	ILVLFVA	0.941
2	9	WILVLFV	0.934
3	12	VLFVAMW	0.925
4	11	LVLFVAM	0.923
5	250	LIFLIVG	0.919
6	13	LFVAMWS	0.918
7	251	IFLIVG	0.898
8	182	CVNITVK	0.898
9	120	AAGAVVG	0.896
10	14	FVAMWSD	0.893
11	249	FLIFLIV	0.886
12	8	SWILVLF	0.881
13	174	NQNNFVH	0.876
14	233	ASVILFS	0.867
15	243	VILLISF	0.856
16	247	ISFLIFL	0.853
17	121	AGAVVGG	0.852
18	7	GSWILVL	0.851
19	123	AVVGGLG	0.832
20	115	VAGAAAA	0.826
21	119	AAAGAVV	0.826
22	252	FLIVG	0.814
23	235	VILFSSP	0.808
24	234	SVILFSS	0.808
25	6	IGSWILV	0.805
26	246	LISFLIF	0.801
27	244	ILLISFL	0.801
28	122	GAVVGGL	0.793
29	175	QNNFVHD	0.792
30	248	SFLIFLI	0.792
31	232	GASVILF	0.777
32	124	VVGGLGG	0.768

33	212	VVEQMCI	0.729
34	245	LLISFLI	0.725
35	116	AGAAAAG	0.721
36	118	AAAAGAV	0.721
37	5	HIGSWIL	0.714
38	173	SNQNNFV	0.714
39	114	HVAGAAA	0.711
40	217	CITQYQR	0.678
41	216	MCITQYQ	0.676
42	178	FVHDCVN	0.665
43	215	QMCITQY	0.661
44	213	VEQMCIT	0.660
45	172	YSNQNNF	0.658
46	225	SQAYYQR	0.650
47	141	LIHFGND	0.647
48	117	GAAAAGA	0.633
49	15	VAMWSDV	0.613
50	16	AMWSDVG	0.613
51	179	VHDCVNI	0.606
52	131	YMLGSAM	0.552
53	4	SHIGSWI	0.550
54	125	VGGLGGY	0.546
55	98	SHSQWNK	0.545
56	132	MLGSAMS	0.532
57	181	DCVNITV	0.531
58	55	QGGGGWG	0.530
59	92	GWGQGGS	0.530
60	91	GGWGQGG	0.530
61	81	GGGWGQP	0.530
62	73	GGGWGQP	0.530
63	57	GGGWGQP	0.530
64	90	GGGWGQG	0.530
65	180	HDCVNIT	0.526
66	93	WGQGGSH	0.523
67	128	LGGYMLG	0.522
68	17	MWSDVGL	0.513
69	177	NFVHDCV	0.511

70	183	VNITVKQ	0.509
71	18	WSDVGLC	0.506
72	176	NNFVHDC	0.501
73	242	PVILLIS	0.497
74	33	GWNTGGS	0.491
75	32	GGWNTGG	0.491
76	31	GGGWNTG	0.491
77	34	WNTGGSR	0.484
78	127	GLGGYML	0.458
79	129	GGYMLGS	0.458
80	89	GGGGWGQ	0.456
81	56	GGGGWGQ	0.456
82	97	GSHSQWN	0.454
83	133	LGSAMSR	0.453
84	130	GYMLGSA	0.450
85	162	NQVYYRP	0.414
86	80	HGGGWGQ	0.396
87	72	HGGGWGQ	0.396
88	88	HGGGGWG	0.396
89	126	GGLGGYM	0.387
90	19	SDVGLCK	0.384
91	160	YPNQVYY	0.378
92	161	PNQVYYR	0.378
93	214	EQMCITQ	0.360
94	224	ESQAYYQ	0.350
95	184	NITVKQH	0.337
96	231	RGASVIL	0.335
97	142	IHFGNDY	0.334
98	189	QHTVTTT	0.332
99	208	IMERVVE	0.330
100	155	ENMYHYP	0.292
101	187	VKQHTVT	0.276
102	111	NMKHVAG	0.273
103	143	HFGNDYE	0.267
104	226	QAYYQRG	0.265
105	94	GQGGSHS	0.257
106	230	QRGASVI	0.255

107	95	QGGSHSQ	0.251
108	96	GGSHSQW	0.251
109	211	RVVEQMC	0.251
110	1	MVKSHIG	0.249
111	159	HYPNQVY	0.248
112	191	TVTTTTK	0.231
113	190	HTVTTTT	0.227
114	113	KHVAGAA	0.226
115	185	ITVKQHT	0.216
116	228	YYQRGAS	0.211
117	227	AYYQRGA	0.211
118	218	ITQYQRE	0.208
119	112	MKHVAGA	0.206
120	171	RYSNQNN	0.200
121	2	VKSHIGS	0.199
122	236	ILFSSPP	0.198
123	99	HSQWNKP	0.180
124	140	PLIHFGN	0.170
125	110	TNMKHVA	0.166
126	30	PGGGWNT	0.164
127	82	GGWGQPH	0.154
128	74	GGWGQPH	0.154
129	58	GGWGQPH	0.154
130	144	FGNDYED	0.154
131	229	YQRGASV	0.153
132	158	YHYPNQV	0.148
133	156	NMYHYPN	0.146
134	157	MYHYPNQ	0.146
135	164	VYYRPVD	0.139
136	84	WGQPHGG	0.125
137	76	WGQPHGG	0.125
138	60	WGQPHGG	0.125
139	83	GWGQPHG	0.125
140	75	GWGQPHG	0.125
141	59	GWGQPHG	0.125
142	169	VDRYSNQ	0.111
143	134	GSAMSRP	0.107

144	20	DVGLCKK	0.103
145	186	TVKQHTV	0.100
146	188	KQHTVTT	0.100
147	198	GENFTET	0.099
148	201	FTETDIK	0.099
149	79	PHGGGWG	0.096
150	71	PHGGGWG	0.096
151	41	YPGQGSP	0.094
152	152	YYRENMY	0.090
153	153	YRENMYH	0.090
154	210	ERVVEQM	0.089
155	209	MERVVEQ	0.088
156	204	TDIKIME	0.082
157	199	ENFTETD	0.081
158	3	KSHIGSW	0.081
159	241	PPVILLI	0.075
160	200	NFTETDI	0.074
161	163	QVYYRPV	0.073
162	45	GSPGGNR	0.071
163	54	PQGGGGW	0.069
164	44	QGSPGGN	0.067
165	43	GQGSPGG	0.067
166	35	NTGGSRY	0.066
167	36	TGGSRYP	0.066
168	223	RESQAYY	0.065
169	192	VTTTTKG	0.063
170	222	QRESQAY	0.061
171	221	YQRESQA	0.059
172	220	QYQRESQ	0.059
173	170	DRYSNQN	0.058
174	240	SPPVILL	0.057
175	78	QPHGGGW	0.055
176	70	QPHGGGW	0.055
177	86	QPHGGGG	0.055
178	61	GQPHGGS	0.055
179	85	GQPHGGG	0.055
180	77	GQPHGGG	0.055

181	21	VGLCKKR	0.054
182	62	QPHGGSR	0.053
183	154	RENMYHY	0.051
184	219	TQYQRES	0.049
185	196	TKGENFT	0.048
186	197	KGENFTE	0.048
187	100	SQWNKPR	0.044
188	239	SSPPVIL	0.044
189	205	DIKIMER	0.042
190	87	PHGGGGW	0.041
191	238	FSSPPVI	0.041
192	139	RPLIHFG	0.037
193	145	GNDYEDR	0.033
194	137	MSRPLIH	0.033
195	207	KIMERVV	0.031
196	65	GGSRDQP	0.029
197	148	YEDRYYR	0.026
198	203	ETDIKIM	0.025
199	136	AMSRPLI	0.025
200	193	TTTTKGE	0.024
201	29	KPGGGWN	0.024
202	206	IKIMERV	0.023
203	195	TTKGENF	0.021
204	47	PGGNRYP	0.021
205	48	GGNRYPP	0.021
206	165	YYRPVDR	0.020
207	69	DQPHGGG	0.020
208	237	LFSSPPV	0.020
209	138	SRPLIHF	0.020
210	40	RYPGQGS	0.020
211	135	SAMSRPL	0.019
212	39	SRYPGQG	0.019
213	52	YPPQGGG	0.017
214	64	HGGSRDQ	0.017
215	46	SPGGNRY	0.014
216	38	GSRYPGQ	0.014
217	37	GGSRYPG	0.014

218	168	PVDRYSN	0.014
219	151	RYYRENM	0.014
220	109	KTNMKHV	0.013
221	53	PPQGGGG	0.012
222	194	TTTKGEN	0.012
223	42	PGQGSPG	0.011
224	202	TETDIKI	0.010
225	101	QWNKPRK	0.009
226	63	PHGGSRD	0.008
227	146	NDYEDRY	0.006
228	147	DYEDRYY	0.006
229	66	GSRDQPH	0.005
230	50	NRYPPQG	0.005
231	22	GLCKKRP	0.004
232	68	RDQPHGG	0.004
233	67	SRDQPHG	0.004
234	49	GNRYPPQ	0.003
235	149	EDRYYRE	0.003
236	150	DRYYREN	0.003
237	51	RYPPQGG	0.003
238	166	YRPVDRY	0.003
239	167	RPVDRYS	0.003
240	108	PKTNMKH	0.003
241	107	KPKTNMK	0.003
242	28	PKPGGGW	0.002
243	102	WNKPRKP	0.001
244	23	LCKKRPK	0.001
245	106	RKPKTNM	0.000
246	27	RPKPGGG	0.000
247	24	CKKRPKP	0.000
248	103	NKPRKPK	0.000
249	26	KRPKPGG	0.000
250	105	PRKPKTN	0.000
251	104	KPRKPKT	0.000
252	25	KKRPKPG	0.000

Table 3 Ligand binding affinity probability

Wild Type	Variant
r=MET;n=1 :prob=0.026	r=MET;n=1 :prob=0.026
r=VAL;n=2 :prob=0.026	r=VAL;n=2 :prob=0.026
r=LYS;n=3 :prob=0.027	r=LYS;n=3 :prob=0.027
r=SER;n=4 :prob=0.026	r=SER;n=4 :prob=0.026
r=HIS;n=5 :prob=0.027	r=HIS;n=5 :prob=0.026
r=ILE;n=6 :prob=0.026	r=ILE;n=6 :prob=0.026
r=GLY;n=7 :prob=0.026	r=GLY;n=7 :prob=0.026
r=SER;n=8 :prob=0.027	r=SER;n=8 :prob=0.026
r=TRP;n=9 :prob=0.031	r=TRP;n=9 :prob=0.026
r=ILE;n=10 :prob=0.028	r=ILE;n=10 :prob=0.027
r=LEU;n=11 :prob=0.027	r=LEU;n=11 :prob=0.026
r=VAL;n=12 :prob=0.027	r=VAL;n=12 :prob=0.027
r=LEU;n=13 :prob=0.026	r=LEU;n=13 :prob=0.027
r=PHE;n=14 :prob=0.027	r=PHE;n=14 :prob=0.032
r=VAL;n=15 :prob=0.030	r=VAL;n=15 :prob=0.027
r=ALA;n=16 :prob=0.028	r=ALA;n=16 :prob=0.026
r=MET;n=17 :prob=0.027	r=MET;n=17 :prob=0.026
r=TRP;n=18 :prob=0.026	r=TRP;n=18 :prob=0.027
r=SER;n=19 :prob=0.026	r=SER;n=19 :prob=0.026
r=ASP;n=20 :prob=0.027	r=ASP;n=20 :prob=0.026
r=VAL;n=21 :prob=0.033	r=VAL;n=21 :prob=0.026
r=GLY;n=22 :prob=0.037	r=GLY;n=22 :prob=0.027
r=LEU;n=23 :prob=0.035	r=LEU;n=23 :prob=0.026
r=CYS;n=24 :prob=0.029	r=CYS;n=24 :prob=0.026
r=LYS;n=25 :prob=0.035	r=LYS;n=25 :prob=0.026
r=LYS;n=26 :prob=0.027	r=LYS;n=26 :prob=0.026
r=ARG;n=27 :prob=0.026	r=ARG;n=27 :prob=0.026
r=PRO;n=28 :prob=0.026	r=PRO;n=28 :prob=0.026
r=LYS;n=29 :prob=0.026	r=LYS;n=29 :prob=0.027
r=PRO;n=30 :prob=0.026	r=PRO;n=30 :prob=0.026
r=GLY;n=31 :prob=0.026	r=GLY;n=31 :prob=0.026
r=GLY;n=32 :prob=0.027	r=GLY;n=32 :prob=0.026
r=GLY;n=33 :prob=0.026	r=GLY;n=33 :prob=0.026
r=TRP;n=34 :prob=0.026	r=TRP;n=34 :prob=0.026
r=ASN;n=35 :prob=0.027	r=ASN;n=35 :prob=0.026
r=THR;n=36 :prob=0.026	r=THR;n=36 :prob=0.026
r=GLY;n=37 :prob=0.026	r=GLY;n=37 :prob=0.026
r=GLY;n=38 :prob=0.026	r=GLY;n=38 :prob=0.026
r=SER;n=39 :prob=0.027	r=SER;n=39 :prob=0.026
r=ARG;n=40 :prob=0.030	r=ARG;n=40 :prob=0.026
r=TYR;n=41 :prob=0.033	r=TYR;n=41 :prob=0.026

r=PRO;n=42 :prob=0.042	r=PRO;n=42 :prob=0.026
r=GLY;n=43 :prob=0.037	r=GLY;n=43 :prob=0.026
r=GLN;n=44 :prob=0.030	r=GLN;n=44 :prob=0.026
r=GLY;n=45 :prob=0.029	r=GLY;n=45 :prob=0.026
r=SER;n=46 :prob=0.027	r=SER;n=46 :prob=0.027
r=PRO;n=47 :prob=0.027	r=PRO;n=47 :prob=0.027
r=GLY;n=48 :prob=0.027	r=GLY;n=48 :prob=0.027
r=GLY;n=49 :prob=0.026	r=GLY;n=49 :prob=0.026
r=ASN;n=50 :prob=0.026	r=ASN;n=50 :prob=0.026
r=ARG;n=51 :prob=0.027	r=ARG;n=51 :prob=0.028
r=TYR;n=52 :prob=0.027	r=TYR;n=52 :prob=0.033
r=PRO;n=53 :prob=0.026	r=PRO;n=53 :prob=0.032
r=PRO;n=54 :prob=0.026	r=PRO;n=54 :prob=0.033
r=GLN;n=55 :prob=0.026	r=GLN;n=55 :prob=0.027
r=GLY;n=56 :prob=0.026	r=GLY;n=56 :prob=0.027
r=GLY;n=57 :prob=0.026	r=GLY;n=57 :prob=0.028
r=GLY;n=58 :prob=0.027	r=GLY;n=58 :prob=0.027
r=GLY;n=59 :prob=0.030	r=GLY;n=59 :prob=0.029
r=TRP;n=60 :prob=0.032	r=TRP;n=60 :prob=0.028
r=GLY;n=61 :prob=0.039	r=GLY;n=61 :prob=0.027
r=GLN;n=62 :prob=0.033	r=GLN;n=62 :prob=0.026
r=PRO;n=63 :prob=0.028	r=PRO;n=63 :prob=0.026
r=HIS;n=64 :prob=0.026	r=HIS;n=64 :prob=0.026
r=GLY;n=65 :prob=0.026	r=GLY;n=65 :prob=0.026
r=GLY;n=66 :prob=0.026	r=GLY;n=66 :prob=0.026
r=GLY;n=67 :prob=0.027	r=SER;n=67 :prob=0.026
r=TRP;n=68 :prob=0.038	r=ARG;n=68 :prob=0.026
r=GLY;n=69 :prob=0.026	r=ASP;n=69 :prob=0.026
r=GLN;n=70 :prob=0.026	r=GLN;n=70 :prob=0.026
r=PRO;n=71 :prob=0.026	r=PRO;n=71 :prob=0.026
r=HIS;n=72 :prob=0.026	r=HIS;n=72 :prob=0.026
r=GLY;n=73 :prob=0.026	r=GLY;n=73 :prob=0.026
r=GLY;n=74 :prob=0.026	r=GLY;n=74 :prob=0.026
r=GLY;n=75 :prob=0.026	r=GLY;n=75 :prob=0.026
r=TRP;n=76 :prob=0.026	r=TRP;n=76 :prob=0.026
r=GLY;n=77 :prob=0.026	r=GLY;n=77 :prob=0.029
r=GLN;n=78 :prob=0.026	r=GLN;n=78 :prob=0.027
r=PRO;n=79 :prob=0.026	r=PRO;n=79 :prob=0.030
r=HIS;n=80 :prob=0.028	r=HIS;n=80 :prob=0.030
r=GLY;n=81 :prob=0.028	r=GLY;n=81 :prob=0.034
r=GLY;n=82 :prob=0.029	r=GLY;n=82 :prob=0.035
r=GLY;n=83 :prob=0.028	r=GLY;n=83 :prob=0.033
r=TRP;n=84 :prob=0.028	r=TRP;n=84 :prob=0.030
r=GLY;n=85 :prob=0.026	r=GLY;n=85 :prob=0.031

r=GLN;n=86 :prob=0.027	r=GLN;n=86 :prob=0.034
r=PRO;n=87 :prob=0.026	r=PRO;n=87 :prob=0.027
r=HIS;n=88 :prob=0.026	r=HIS;n=88 :prob=0.030
r=GLY;n=89 :prob=0.026	r=GLY;n=89 :prob=0.026
r=GLY;n=90 :prob=0.026	r=GLY;n=90 :prob=0.026
r=GLY;n=91 :prob=0.026	r=GLY;n=91 :prob=0.026
r=GLY;n=92 :prob=0.026	r=GLY;n=92 :prob=0.026
r=TRP;n=93 :prob=0.026	r=TRP;n=93 :prob=0.026
r=GLY;n=94 :prob=0.026	r=GLY;n=94 :prob=0.026
r=GLN;n=95 :prob=0.026	r=GLN;n=95 :prob=0.026
r=GLY;n=96 :prob=0.026	r=GLY;n=96 :prob=0.026
r=GLY;n=97 :prob=0.026	r=GLY;n=97 :prob=0.026
r=SER;n=98 :prob=0.026	r=SER;n=98 :prob=0.026
r=HIS;n=99 :prob=0.026	r=HIS;n=99 :prob=0.026
r=SER;n=100 :prob=0.026	r=SER;n=100 :prob=0.027
r=GLN;n=101 :prob=0.026	r=GLN;n=101 :prob=0.028
r=TRP;n=102 :prob=0.026	r=TRP;n=102 :prob=0.030
r=ASN;n=103 :prob=0.027	r=ASN;n=103 :prob=0.032
r=LYS;n=104 :prob=0.027	r=LYS;n=104 :prob=0.033
r=PRO;n=105 :prob=0.029	r=PRO;n=105 :prob=0.034
r=SER;n=106 :prob=0.028	r=ARG;n=106 :prob=0.031
r=LYS;n=107 :prob=0.028	r=LYS;n=107 :prob=0.032
r=PRO;n=108 :prob=0.027	r=PRO;n=108 :prob=0.030
r=LYS;n=109 :prob=0.026	r=LYS;n=109 :prob=0.029
r=THR;n=110 :prob=0.026	r=THR;n=110 :prob=0.030
r=ASN;n=111 :prob=0.026	r=ASN;n=111 :prob=0.028
r=MET;n=112 :prob=0.026	r=MET;n=112 :prob=0.027
r=LYS;n=113 :prob=0.026	r=LYS;n=113 :prob=0.027
r=HIS;n=114 :prob=0.026	r=HIS;n=114 :prob=0.027
r=VAL;n=115 :prob=0.026	r=VAL;n=115 :prob=0.027
r=ALA;n=116 :prob=0.026	r=ALA;n=116 :prob=0.027
r=GLY;n=117 :prob=0.026	r=GLY;n=117 :prob=0.026
r=ALA;n=118 :prob=0.026	r=ALA;n=118 :prob=0.027
r=ALA;n=119 :prob=0.027	r=ALA;n=119 :prob=0.027
r=ALA;n=120 :prob=0.029	r=ALA;n=120 :prob=0.026
r=ALA;n=121 :prob=0.027	r=ALA;n=121 :prob=0.026
r=GLY;n=122 :prob=0.026	r=GLY;n=122 :prob=0.027
r=ALA;n=123 :prob=0.027	r=ALA;n=123 :prob=0.027
r=VAL;n=124 :prob=0.028	r=VAL;n=124 :prob=0.027
r=VAL;n=125 :prob=0.042	r=VAL;n=125 :prob=0.069
r=GLY;n=126 :prob=0.028	r=GLY;n=126 :prob=0.030
r=GLY;n=127 :prob=0.030	r=GLY;n=127 :prob=0.030
r=LEU;n=128 :prob=0.034	r=LEU;n=128 :prob=0.066
r=GLY;n=129 :prob=0.041	r=GLY;n=129 :prob=0.064

r=GLY;n=130 :prob=0.034
r=TYR;n=131 :prob=0.047
r=MET;n=132 :prob=0.037
r=LEU;n=133 :prob=0.040
r=GLY;n=134 :prob=0.030
r=SER;n=135 :prob=0.027
r=ALA;n=136 :prob=0.027
r=MET;n=137 :prob=0.026
r=SER;n=138 :prob=0.026
r=ARG;n=139 :prob=0.026
r=PRO;n=140 :prob=0.026
r=LEU;n=141 :prob=0.026
r=ILE;n=142 :prob=0.027
r=HIS;n=143 :prob=0.026
r=PHE;n=144 :prob=0.026
r=GLY;n=145 :prob=0.026
r=ASN;n=146 :prob=0.026
r=ASP;n=147 :prob=0.026
r=TYR;n=148 :prob=0.026
r=GLU;n=149 :prob=0.027
r=ASP;n=150 :prob=0.026
r=ARG;n=151 :prob=0.026
r=TYR;n=152 :prob=0.027
r=TYR;n=153 :prob=0.027
r=ARG;n=154 :prob=0.026
r=GLU;n=155 :prob=0.026
r=ASN;n=156 :prob=0.027
r=MET;n=157 :prob=0.026
r=TYR;n=158 :prob=0.026
r=ARG;n=159 :prob=0.026
r=TYR;n=160 :prob=0.026
r=PRO;n=161 :prob=0.026
r=ASN;n=162 :prob=0.026
r=GLN;n=163 :prob=0.029
r=VAL;n=164 :prob=0.028
r=TYR;n=165 :prob=0.038
r=TYR;n=166 :prob=0.029
r=ARG;n=167 :prob=0.028
r=PRO;n=168 :prob=0.029
r=VAL;n=169 :prob=0.026
r=ASP;n=170 :prob=0.027
r=ARG;n=171 :prob=0.026
r=TYR;n=172 :prob=0.026
r=SER;n=173 :prob=0.026

r=GLY;n=130 :prob=0.030
r=TYR;n=131 :prob=0.071
r=MET;n=132 :prob=0.031
r=LEU;n=133 :prob=0.031
r=GLY;n=134 :prob=0.028
r=SER;n=135 :prob=0.026
r=ALA;n=136 :prob=0.026
r=MET;n=137 :prob=0.026
r=SER;n=138 :prob=0.026
r=ARG;n=139 :prob=0.026
r=PRO;n=140 :prob=0.026
r=LEU;n=141 :prob=0.026
r=ILE;n=142 :prob=0.026
r=HIS;n=143 :prob=0.026
r=PHE;n=144 :prob=0.026
r=GLY;n=145 :prob=0.026
r=ASN;n=146 :prob=0.026
r=ASP;n=147 :prob=0.026
r=TYR;n=148 :prob=0.026
r=GLU;n=149 :prob=0.026
r=ASP;n=150 :prob=0.026
r=ARG;n=151 :prob=0.026
r=TYR;n=152 :prob=0.031
r=TYR;n=153 :prob=0.026
r=ARG;n=154 :prob=0.026
r=GLU;n=155 :prob=0.026
r=ASN;n=156 :prob=0.026
r=MET;n=157 :prob=0.026
r=TYR;n=158 :prob=0.026
r=HIS;n=159 :prob=0.026
r=TYR;n=160 :prob=0.026
r=PRO;n=161 :prob=0.026
r=ASN;n=162 :prob=0.026
r=GLN;n=163 :prob=0.027
r=VAL;n=164 :prob=0.027
r=TYR;n=165 :prob=0.063
r=TYR;n=166 :prob=0.027
r=ARG;n=167 :prob=0.026
r=PRO;n=168 :prob=0.026
r=VAL;n=169 :prob=0.026
r=ASP;n=170 :prob=0.026
r=ARG;n=171 :prob=0.026
r=TYR;n=172 :prob=0.026
r=SER;n=173 :prob=0.026

r=ASN;n=174 :prob=0.026
r=GLN;n=175 :prob=0.026
r=ASN;n=176 :prob=0.026
r=ASN;n=177 :prob=0.026
r=PHE;n=178 :prob=0.026
r=VAL;n=179 :prob=0.026
r=HIS;n=180 :prob=0.026
r=ASP;n=181 :prob=0.026
r=CYS;n=182 :prob=0.026
r=VAL;n=183 :prob=0.026
r=ASN;n=184 :prob=0.026
r=ILE;n=185 :prob=0.039
r=THR;n=186 :prob=0.026
r=VAL;n=187 :prob=0.026
r=LYS;n=188 :prob=0.031
r=GLN;n=189 :prob=0.041
r=HIS;n=190 :prob=0.026
r=THR;n=191 :prob=0.026
r=VAL;n=192 :prob=0.031
r=THR;n=193 :prob=0.031
r=THR;n=194 :prob=0.026
r=THR;n=195 :prob=0.026
r=THR;n=196 :prob=0.026
r=LYS;n=197 :prob=0.026
r=GLY;n=198 :prob=0.026
r=GLU;n=199 :prob=0.026
r=ASN;n=200 :prob=0.026
r=PHE;n=201 :prob=0.026
r=THR;n=202 :prob=0.026
r=GLU;n=203 :prob=0.026
r=THR;n=204 :prob=0.026
r=ASP;n=205 :prob=0.026
r=ILE;n=206 :prob=0.045
r=LYS;n=207 :prob=0.510
r=ILE;n=208 :prob=0.046
r=MET;n=209 :prob=0.027
r=GLU;n=210 :prob=0.118
r=ARG;n=211 :prob=0.045
r=VAL;n=212 :prob=0.026
r=VAL;n=213 :prob=0.026
r=GLU;n=214 :prob=0.026
r=GLN;n=215 :prob=0.026
r=MET;n=216 :prob=0.026
r=CYS;n=217 :prob=0.026

r=ASN;n=174 :prob=0.026
r=GLN;n=175 :prob=0.026
r=ASN;n=176 :prob=0.026
r=ASN;n=177 :prob=0.026
r=PHE;n=178 :prob=0.026
r=VAL;n=179 :prob=0.026
r=HIS;n=180 :prob=0.026
r=ASP;n=181 :prob=0.026
r=CYS;n=182 :prob=0.026
r=VAL;n=183 :prob=0.026
r=ASN;n=184 :prob=0.026
r=ILE;n=185 :prob=0.116
r=THR;n=186 :prob=0.026
r=VAL;n=187 :prob=0.026
r=LYS;n=188 :prob=0.058
r=GLN;n=189 :prob=0.510
r=HIS;n=190 :prob=0.027
r=THR;n=191 :prob=0.026
r=VAL;n=192 :prob=0.058
r=THR;n=193 :prob=0.058
r=THR;n=194 :prob=0.026
r=THR;n=195 :prob=0.026
r=THR;n=196 :prob=0.026
r=LYS;n=197 :prob=0.026
r=GLY;n=198 :prob=0.026
r=GLU;n=199 :prob=0.026
r=ASN;n=200 :prob=0.026
r=PHE;n=201 :prob=0.026
r=THR;n=202 :prob=0.026
r=GLU;n=203 :prob=0.026
r=THR;n=204 :prob=0.026
r=ASP;n=205 :prob=0.026
r=ILE;n=206 :prob=0.026
r=LYS;n=207 :prob=0.026
r=ILE;n=208 :prob=0.036
r=MET;n=209 :prob=0.026
r=GLU;n=210 :prob=0.026
r=ARG;n=211 :prob=0.031
r=VAL;n=212 :prob=0.036
r=VAL;n=213 :prob=0.026
r=GLU;n=214 :prob=0.026
r=GLN;n=215 :prob=0.026
r=MET;n=216 :prob=0.026
r=CYS;n=217 :prob=0.026

r=ILE;n=218 :prob=0.026
r=THR;n=219 :prob=0.026
r=GLN;n=220 :prob=0.026
r=TYR;n=221 :prob=0.026
r=GLN;n=222 :prob=0.026
r=ARG;n=223 :prob=0.026
r=GLU;n=224 :prob=0.026
r=SER;n=225 :prob=0.026
r=GLN;n=226 :prob=0.026
r=ALA;n=227 :prob=0.026
r=TYR;n=228 :prob=0.026
r=TYR;n=229 :prob=0.026
r=GLN;n=230 :prob=0.026
r=ARG;n=231 :prob=0.026
r=GLY;n=232 :prob=0.026
r=ALA;n=233 :prob=0.026
r=SER;n=234 :prob=0.026
r=VAL;n=235 :prob=0.026
r=ILE;n=236 :prob=0.026
r=LEU;n=237 :prob=0.026
r=PHE;n=238 :prob=0.026
r=SER;n=239 :prob=0.026
r=SER;n=240 :prob=0.026
r=PRO;n=241 :prob=0.026
r=PRO;n=242 :prob=0.026
r=VAL;n=243 :prob=0.026
r=ILE;n=244 :prob=0.026
r=LEU;n=245 :prob=0.026
r=LEU;n=246 :prob=0.026
r=ILE;n=247 :prob=0.026
r=SER;n=248 :prob=0.026
r=PHE;n=249 :prob=0.026
r=LEU;n=250 :prob=0.026
r=ILE;n=251 :prob=0.026
r=PHE;n=252 :prob=0.026
r=LEU;n=253 :prob=0.026
r=ILE;n=254 :prob=0.026
r=VAL;n=255 :prob=0.026
r=GLY;n=256 :prob=0.026

r=ILE;n=218 :prob=0.026
r=THR;n=219 :prob=0.026
r=GLN;n=220 :prob=0.026
r=TYR;n=221 :prob=0.027
r=GLN;n=222 :prob=0.026
r=ARG;n=223 :prob=0.026
r=GLU;n=224 :prob=0.026
r=SER;n=225 :prob=0.027
r=GLN;n=226 :prob=0.026
r=ALA;n=227 :prob=0.026
r=TYR;n=228 :prob=0.026
r=TYR;n=229 :prob=0.026
r=GLN;n=230 :prob=0.026
r=ARG;n=231 :prob=0.026
r=GLY;n=232 :prob=0.026
r=ALA;n=233 :prob=0.026
r=SER;n=234 :prob=0.026
r=VAL;n=235 :prob=0.026
r=ILE;n=236 :prob=0.026
r=LEU;n=237 :prob=0.026
r=PHE;n=238 :prob=0.026
r=SER;n=239 :prob=0.026
r=SER;n=240 :prob=0.026
r=PRO;n=241 :prob=0.026
r=PRO;n=242 :prob=0.026
r=VAL;n=243 :prob=0.026
r=ILE;n=244 :prob=0.026
r=LEU;n=245 :prob=0.026
r=LEU;n=246 :prob=0.026
r=ILE;n=247 :prob=0.026
r=SER;n=248 :prob=0.026
r=PHE;n=249 :prob=0.026
r=LEU;n=250 :prob=0.026
r=ILE;n=251 :prob=0.026
r=PHE;n=252 :prob=0.026
r=LEU;n=253 :prob=0.026
r=ILE;n=254 :prob=0.027
r=VAL;n=255 :prob=0.026
r=GLY;n=256 :prob=0.026

Metal Binding sites UniProtKB - P52113

Position/ codon	
65	
66	
72	
73	
74	
80	
81	
82	
88	
90	
91	
